# Exploratory analysis of real personal emergency response call conversations: considerations for personal emergency response spoken dialogue systems

**DOI:** 10.1186/s12984-016-0207-9

**Published:** 2016-11-14

**Authors:** Victoria Young, Elizabeth Rochon, Alex Mihailidis

**Affiliations:** 1 Rehabilitation Sciences Institute, University of Toronto, Toronto, ON Canada; 2 Institute of Biomaterials and Biomedical Engineering, University of Toronto, Toronto, ON Canada; 3 University Health Network - Toronto Rehabilitation Institute, Toronto, ON Canada; 4 Rehabilitation Sciences Institute, Department of Speech-Language Pathology, University of Toronto, Toronto, ON Canada; 5 Department of Occupational Science and Occupational Therapy, University of Toronto, Toronto, ON Canada

**Keywords:** Personal emergency response system, Conversation analysis, Artificial intelligence, Communication, Aging-in-place, Human-computer interaction, Dialogue planning

## Abstract

**Background:**

The purpose of this study was to derive data from real, recorded, personal emergency response call conversations to help improve the artificial intelligence and decision making capability of a spoken dialogue system in a smart personal emergency response system. The main study objectives were to: develop a model of personal emergency response; determine categories for the model’s features; identify and calculate measures from call conversations (verbal ability, conversational structure, timing); and examine conversational patterns and relationships between measures and model features applicable for improving the system’s ability to automatically identify call model categories and predict a target response.

**Methods:**

This study was exploratory and used mixed methods. Personal emergency response calls were pre-classified according to call model categories identified qualitatively from response call transcripts. The relationships between six verbal ability measures, three conversational structure measures, two timing measures and three independent factors: caller type, risk level, and speaker type, were examined statistically.

**Results:**

Emergency medical response services were the preferred response for the majority of medium and high risk calls for both caller types. Older adult callers mainly requested non-emergency medical service responders during medium risk situations. By measuring the number of spoken words-per-minute and turn-length-in-words for the first spoken utterance of a call, older adult and care provider callers could be identified with moderate accuracy. Average call taker response time was calculated using the number-of-speaker-turns and time-in-seconds measures. Care providers and older adults used different conversational strategies when responding to call takers. The words ‘ambulance’ and ‘paramedic’ may hold different latent connotations for different callers.

**Conclusions:**

The data derived from the real personal emergency response recordings may help a spoken dialogue system classify incoming calls by caller type with moderate probability shortly after the initial caller utterance. Knowing the caller type, the target response for the call may be predicted with some degree of probability and the output dialogue could be tailored to this caller type. The average call taker response time measured from real calls may be used to limit the conversation length in a spoken dialogue system before defaulting to a live call taker.

## Background

This study is part of a larger project involving the design and development of a smart home health monitoring system called, the Health Evaluation Logging and Personal Emergency Response (HELPER) system. The HELPER system incorporates automatic fall detection and a spoken dialogue system (SDS) for contacting emergency assistance into a smart home. This system is further described by [1–4]. A prototype SDS for personal emergency response (PER) was successfully developed for the HELPER system but the user vocabulary was limited to only two words “yes” or “no” [3, 4]. The HELPER SDS has also only been tested with younger adults in simulated emergency situations. Continuing from this previous work, this study sought to identify data useful for improving the robustness of the SDS prior to field testing with older adult users.

During a PER call, a live PER call taker is able to identify the main caller, assess the situation risk, modify on-going dialogue, and determine the desired or appropriate response. The overall goal of this study was to conduct exploratory content analyses on real, transcribed PER call conversations to derive data that could be used to improve a SDS’s ability to artificially mimic the intelligence and decision making capability of a human call taker.

### Smart homes and personal emergency response systems

In recent decades there has been mounting concern over the rising aging population, with associated long term care requirements threatening to overwhelm an already heavily burdened healthcare system. As a result, greater emphasis is being placed on the use of assistive technologies to help support “aging-in-place” outside of the healthcare institution. Of particular interest to this study is an assistive technology called the personal emergency response system (PERS) or care alarm that functions by connecting users who need personal emergency assistance to a live PER operator, any time of the day or night, at the push of a body worn “button” (i.e., necklace, pendant, or watch) [5–7]. A PERS is primarily used by older adults who have mobility difficulties and/or who are at higher risk for medical complications (e.g., frail elderly with multi-morbidities) but who wish to remain living at home. Despite demonstrated system benefits, including (1) reduced anxiety in PERS subscribers and providers and (2) lower overall health care costs [7–9], many potential older adult subscribers are resistant to PERS adoption [6, 10]. Additionally, amongst PERS subscribers, a significant number do not actually “push the button” during a true situation of need [11–14]. To help overcome these barriers, researchers and technology developers are looking at ways to better design the traditional push-button PERS [13, 15–17].

With recent advances in telecommunications and information and computing technologies, a new generation of “smart” PERS is being designed that can be embedded into other “smart” technology platforms, such as smart phones, robots, or smart homes [6]. A “smart home” that incorporates health monitoring is defined as a “residence equipped with technology that enhances the safety of patients at home and monitors their health conditions” [18, 19]. Smart home health systems might, for example, perform continuous home and health monitoring via sensors or cameras strategically positioned within the home [11, 20]. Thus, in the event of an adverse event (e.g. a fall) or an abnormality in the daily living routine being detected, a smart PERS might initiate contact with the user using speech to determine if help is required. The benefits of having a smart home with PER includes: (1) if user is non-responsive and an adverse event is detected the PERS could call for assistance automatically via telephone, text messaging, or email; (2) user could cancel a call before reaching a live person (e.g. false alarm); and (3) continuous user monitoring may provide enough data to detect an adverse event prior to its occurrence and inform the user or care provider that a medical intervention may be required.

### Spoken dialogue systems for personal emergency response

The ability for users to communicate with smart home technologies using only speech via a spoken dialogue system (SDS) has been an area of growing research [21–23]. A SDS is a computer program that engages the user in a conversation by accepting speech input from the user and producing speech output to accomplish a specific goal or task [24]. Essentially mirroring human-to-human turn-taking in a conversation. The feasibility of using speech to activate and control a PERS has been raised by several researchers who found that older adults were receptive to using speech to interact with assistive home technologies including PERS [12, 25, 26]. A study by [12] found that a majority of PERS subscribers would be open to the idea having a PERS automatically call for help in the case of “heavy falls.” Using speech to activate a call could also remove the need to wear a body worn activator, reduce accidental button presses, as well as support user autonomy.

Only a small number of these studies are actually designing and developing a SDSs for PER type situations. Chen et al. [27] designed an automatic speech recognition (ASR) module with limited vocabulary for use in accessing personal emergency assistance by older adults on a mobile cell phone platform. An ASR is a basic component of all SDS [24, 28–30]. Kim et al. [31] proposed a multi-modal approach to PER using inputs from vision, voice, and a body worn gravity sensor. Vacher et al. [32] are working on a ‘smart home’ system called the SWEET HOME that also includes a SDS interface, described by [33], in which personal emergency assistance can be requested. McLean [4] and Hamill et al. [3] are working on the smart PERS component of the HELPER, smart home health monitoring, system.

Although these studies demonstrate it is possible to use a SDS within a PERS, as of this writing, none of these systems were ready to be deployed safely into the real-world and sold on the consumer market. Given the emergency context and the potential for personal injury or harm to life, development and testing must demonstrate that these systems are robust enough for real world situations. They need to be designed especially well to work within real-emergency situations with real end-users and must have built-in contingencies for unexpected inputs (e.g., user cannot be understood despite responding, user doesn’t respond).

### Designing a robust spoken dialogue system

Successful communication between a user and a SDS depends on the system’s ability to both recognize the user’s speech and to adapt to the user’s speaking style (i.e. way of speaking). An individual’s speaking style is heavily influenced by the speaker’s environment, his/her situation, the response from the person(s) being conversed with, and the physical and emotional state of the speaker [34]. The majority of SDSs used in consumer products (e.g., cars, phones) tend to have restricted vocabulary and limited ability to adapt to different speaking styles. Often users are required to modify the way they speak (i.e., well-spaced and clear) and to limit the choice of words they use (i.e., must use key words). When a user experiences repeated conversational difficulties with a SDS, user frustration can soon lead to technology abandonment.

López-Cózar et al. [24] asserts that to design and develop a well-functioning, dynamically adaptable SDS, it is necessary to have correct models of the end-user, and knowledge of the user states. This knowledge may include what utterances (e.g., words, phrases) the user might say (lexical semantics), how (s)he might speak these utterances (e.g., speaking style [34], paralinguistics [35]), the environment where or condition under which the dialogue occurs (situational context), the reason why the words were spoken (speaker intent), and what utterances might be spoken next (prediction) [24]. Designing and developing a device by considering the end-user and the end-user environment is in-line with the theory of “user sensitive inclusive design,” an extension of the user-centred design paradigm. In general, this theory asserts that the design of a technology should consider how various end-users, regardless of age or disability, will interact with the device in their natural environment, may include user feedback during the design and development process, and supports inclusive design to the extent that it does not disadvantage the function of the technology for the intended user [36, 37].

### Spoken dialogue systems and older adult users

As of this writing, only a small number of SDS for use in smart home applications have been tested with older adult users [38]. Collectively, the research indicates that older adult users tend to have greater difficulty adapting their speech when using SDSs, in comparison to younger adult users, and older adult users were found to converse with these systems more as if it were a real human [23, 38, 39]. For example, when conversing with the SDS, the older adult users included more social and polite words such as ‘please’ and ‘thank you’. A few research studies have also examined the use of ASR systems with older adult users [39–42]. Their results have systematically revealed high acoustic variability between older adult and younger adult voices leading to higher word recognition error rates for older adult speakers compared to their younger counterparts [39–42]. Within the older adult cohort itself, there also exists high variability in speech quality and communication ability. This ‘within cohort variability’ depends significantly on the individual’s overall health condition. Typically, the natural process of aging causes physiological changes that affect speech, such as slowing of motor processes and shortening of attention span and memory [43]. Health complications (e.g., stroke, Parkinson’s disease, aphasia, multiple-sclerosis, dementia, hearing impairment) can further affect one’s speech and communication [43]. In emergency or stressful situations, human speech may also become altered to the point of impairment or disorder because of strong emotion, medical trauma, and disease exacerbations [44–48] (p.359). The resulting disordered speech may be observed as slower communication and speech output; incomplete utterances, hesitations; word sound errors; word finding difficulties; and reduced speech amount [43, 49, 50].

Although these speech characteristics or paralinguistic qualities of speech do not affect the lexical semantics or meaning of words, the SDS’s ability to correctly and automatically recognize the incoming speech will often be affected. By definition, paralinguistic communication includes “vocal, but extra-verbal aspects of communication properties, such as voice power (volume), the rate of speaking, variations, errors and other distortions of speech fluency, setting or level of voice, and its quality” [35]. Using paralinguistic communication to our advantage, research studies have shown that measures of paralinguistic speech features could be used to differentiate between older and younger speakers successfully [51] and to aid in the identification of a speaker’s emotional state [52, 53]. Lefter et al. [52] also suggested if a caller’s stress could be measured, emergency call centres could allow computer operators to handle the less stressed callers, while the higher stressed individuals could be handled by a human operator.

### Study rational

In PER events, with non-ideal speakers and potentially stressful or emotional environments, we assumed that speech recognition errors would be inescapable. Existing research literature has shown that even in optimal conditions with “designed-for” end-users the ability for a computer to accurately recognize incoming speech is challenging [39, 54, 55]. However, it may be possible to compensate for some of these errors through intelligent SDS design. For example, by incorporating into the dialogue system routines for error checking and input verification [39, 55, 56]. Non-lexical support could also be leveraged from the call conversation, such as caller verbal ability (i.e., paralinguistic speech features) and conversational structure, to further inform the SDS and support decision making with higher confidence.

As previously mentioned, many older adult users were found to communicate with a SDS as if it were human. Therefore, it was hypothesized that studying real PER call conversations between human PERS users and human call takers could provide the needed knowledge to help design a ‘robust’ or well-functioning and dynamically adaptable SDS for personal emergency situations with older adult callers. These real conversations could be analyzed to identify data for strengthening the design making capability and intelligence of the SDS in a smart PERS, improving its ability to adapt to different end-user needs quickly and effectively. Given that inducing PER call situations is difficult and unethical; and asking previous PERS users to recall how they might have conversed or responded during a prior personal emergency situation may not yield reliable data; it was decided that secondary data in the form of recorded conversations from real, PER calls could be used for this study.

With respect to prior research on PER calls, although previous research studies have analysed recorded and transcribed conversations from North American 911 community emergency response call lines (similar to 112 in Europe or 000 in Australia) [57–62], the paper authors could not locate research studies (in English) that specifically analyzed PER call conversations.

### Study objectives

The main objectives of this study were to: (1) develop a PER model; (2) determine categories for the PER model features; (3) identify and calculate measures from call conversations (verbal ability, conversational structure, timing); and (4) examine conversational patterns and relationships between measures and model features applicable for improving the SDS’s ability to automatically identify categories within the call model and predict a target response.

This study does not include a complete analysis of the lexical semantics of the PER dialogue and does not examine “what” the user utters. A traditional qualitative conversation analysis involving speech act coding at the utterance or conversational level will also not be part of this study analysis. These topics fall outside the scope of this paper.

### Study significance

This study demonstrates how secondary conversational data can be analyzed to derive contextual information that can be applied to or used to inform the design of a SDS. Specifically to improve the artificial intelligence and decision making capability of a SDS for PER events so that it can quickly and effectively converse with the end-user and determine his/her desired, or the best possible, response. These study results may also be useful for other research groups who are developing adaptable SDSs for smart home technologies or assistive robots that interact with older adults in potentially stressful situations. Emergency medical response personnel, clinicians and care providers may also find these study results useful to help them understand communication differences that may arise between PERS users during various personal emergency situations. Additionally, the results might be relevant to the development of personal emergency communication protocols.

### Paper structure

The study methodology will be presented next followed by the results divided into two parts. The first part describes the qualitative portion of the study in which a PER model is developed. The second part describes the quantitative portion of the study and summarizes the results of analyses performed on the transcribed recorded call data. The analyses use select conversational measures and categories from the PER model identified from part one of the study. The paper ends with a discussion on the study findings.

## Methods

### Research design method

This study follows an exploratory, sequential, mixed-methods design using content analyses to examine the transcripts of real, recorded, PER call conversations in Canadian English. The conversations take place between a human call operator (within a PER call centre call (i.e., the call taker) and a human PERS user (i.e. the caller). The PER calls (herein called “calls”) were all acquired from a local PER call centre in Toronto, Canada (herein called “call centre”). The company’s name is withheld for reasons of confidentiality. This design approach begins with a ‘qualitative data collection and analysis’ followed by a ‘quantitative data collection and analysis’ and ends with a ‘final interpretation’ [63].

### Content analysis

Content analysis has been applied in many research fields for analyzing text and other media in context [64, 65]. It is known to be a systematic, objective, and repeatable research method as well as a valid means of quantifying phenomena or making inferences about data in context [64] or for building a model, conceptual system or map [66]. The outcome of a content analysis may be used to guide future action which is especially useful in the field of health research [66]. In the field of artificial intelligence, researchers use content analysis to help design machines capable of understanding natural language [64]. Content analysis is flexible enough to examine data both qualitatively or quantitatively and inductively (e.g., specific to general) or deductively (e.g., general to specific based on existing theory) [64, 66]. However, this flexibility is also its limitation. Some researchers note that because content analysis does not proceed linearly and has minimal formalized procedures, it can become more complex and difficult to implement than quantitative analysis [67].

### Content analysis general procedure

The general procedure for implementing a content analysis is described in [64, 66, 68]:

1. Select a unit of analysis (e.g., interviews, a program, parts of text);
2. Within the unit of analysis, select a meaning/coding/content/recording unit. Essentially, one must decide what to analyse, to what degree of detail, and how sampling will be conducted (e.g., should the codes include silence, sighs, laughter, and postures?);
3. Organize the data (e.g., use open coding, categories, themes, abstractions);
4. Create a model, conceptual system or map, or categories.

### Content analysis approaches

When the content under study is a conversation, as in this case, content analysis becomes a conversation analysis [64]. Conversation analysis has been a technique used since the late 1960’s [69]. Two approaches to applying content analysis are used in this study: (1) a conventional conversational analysis, followed by (2) a quantitative conversational analysis. For a *conventional conversation analysis*, coding categories are typically derived directly from the conversational data and are generally used to describe a phenomenon in the data [70]. For a *quantitative conversation analysis*, conversational data are sorted into explicit categories and then described using statistics [71]. Further details on the conversation analysis method are described by [64, 72, 73].

### Research design details

#### Data collection

##### Personal emergency response call recordings

Digital audio recordings of live PER calls between a real caller and a real call centre call taker were obtained from a local, private PER service provider in Canada. We requested audio samples of both emergency and non-emergency calls. The non-emergency call samples included: false alarms or accidental system activations, installation setups or equipment test calls, scheduled check-ins, translation requests, and follow-up calls. The emergency calls recorded included genuine emergency calls for either Emergency Medical Services (EMS) or non-EMS responders. In Canada, EMS is an organization run by the municipality and is mandated to provide emergency medical response to designated regions in their jurisdiction (e.g., towns, cities). EMS usually includes the paramedic, firefighter, and police responders. Paramedics specifically are the individuals trained to provide medical care or medical assistance during emergencies 24/7, both pre-hospital and, occasionally, “out-of-hospital” for select special needs individuals in certain communities [74]. In this study, non-EMS responders were defined as family relatives, friends, and/or professional care providers (i.e., a paid personal support worker or nurse) who were not paramedics, firefighters or police. A total of 109 digitized call recordings were obtained from the PERS provider (name withheld for confidentiality reasons). These recordings were collected over two years (2008–52 calls and 2009–57 calls). To our knowledge, all clients in this study used the traditional push-button activator. We are unaware of any prior call “sorting”, for example, with respect to gender, call reason, caller type, and emergency risk level that may have occurred.

##### Confidentiality

Confidentiality agreements were signed between the private PER service provider providing the audio recording samples and the Intelligent Assistive Technology and Systems Lab in the Rehabilitation Sciences Institute at the University of Toronto, Canada. These agreements outlined how the data could be used and stored and who had access to the data. Due to the nature of the working agreement with the PERS provider, only a limited number of research team members had permission to listen to the raw call recordings. No identifying information was included in the transcripts (i.e., no names, addresses, or contact information).

##### Research population

The call recordings only included the audio data of the call and did not include any information about the actual call takers who took the call or the callers themselves such as medical conditions, medications, age, gender, ethnicity, or length of time as a PERS user. Additionally, no information was provided about the call takers’ background training, age, gender, or professional experience in the field. Caller age and gender were deduced from the call conversations where possible. Caller gender was postulated based on clues from the conversation (i.e. use of “him” or “her” from a care provider, or perceived voice pitch). If an age was mentioned in the conversation, this number was noted in the comments section of the call transcript. Typically, the majority of PERS users are older adult women [9, 75, 76].

Information on the identity of the call takers speaking to the callers was not provided. However, from listening to the conversations, at least 18 different call takers could be identified by name. In 5 of these calls the call taker’s name was not provided but judging by the voice quality and speaking style, it is believed that none of these callers were outside of the prior 18 identified.

#### Data processing

##### Call transcription and data extraction

Eighty-four (84) response calls were transcribed in total. The 24 non-transcribed calls consisted of repeat recordings or were conversations between the emergency response service providers only (i.e. between the call taker and EMS dispatchers) or PERS setup personnel. Transcription was performed by the first author verbatim from digital audio files using the computer software, “Systematic Analysis of Language Transcripts” (SALT), version 8.0 and 9.0 [77] and Audacity audio software version 2.02 [78]. Transcriptions were performed at the University of Toronto in the Intelligent Assistive Technology and Systems Lab. SALT was selected because this program could be used for transcription of conversations and also because the software had language analysis capability for the measures of interest in this study (e.g., words per minute, sentence type, number of speaker turns, etc.). These measures were very important for the quantitative analysis portion of this study. See the “Data Analysis” section for further information on the specific language measures of interest. The transcription process followed the SALT protocol outlined in the user manual [79]. An example of the transcript can be seen in the “Call Examples” section of the paper. In addition to transcribing the spoken words, a start and end time was calculated for each conversation. The start time began with the first spoken sound or word of the conversation for the first speaker. The end time was marked as the first silence at the end of the last utterance in the conversation. Disfluencies and unintelligible words were marked in the transcripts. Further details are described in the “Data Analysis” section. During transcription, effort was made to capture non-word utterances (e.g., coughing), fillers (e.g., ‘eh’, ‘ah’), and to note moments of conversational silence (e.g., long pauses).

##### Data analysis

The study was divided into two parts. Part 1 looked at the call conversations qualitatively to identify call categories or reoccurring themes that could be used to model a PER event. The PER model was then used to organize the calls into different call categories. Part 2 focused on the quantitative analyses of call measures with call characteristics identified from the PER model. The procedures used to analyse the data are summarized here:

1. PER call recordings were acquired from a private PER service provider;
2. Acquired PER calls were transcribed;
3. “Naïve” listening of the call recordings and reading of the transcripts were performed to obtain a superficial and preliminary understanding of the call conversations and to identify possible directions for analysis;
4. A conventional conversation analysis was performed using the ‘call conversation’ as the coding unit of analysis for the different types of responders (target responses) requested by callers. These different “responder” requests or “target responses” comprise the individuals or type of assistance desired by the caller or PERS user (e.g., an ambulance, or a family member). In this case the “ambulance” and “family member” are two different target responses. Responder requests were further organized into a higher level grouping called “response types.” Response types describe the different target responses for each of the calls. For example, EMS was a response type that includes requests for ambulance and paramedic target responses. The calls were sorted by their target responses and response types.;
5. A model of the PER event was developed, called the PER model, which contains various call categories that make up essential elements of PER events;
6. The collected calls were then organized using the call categories from the PER model;
7. A quantitative conversation analysis was performed next to examine the relationships between various conversational measures and call categories;
8. Various conversational measures were selected and acquired using the transcribed call data and SALT software;
9. The data was statistically analysed using IBM SPSS statistical software package versions 21–23 [80]. Significant relationships between measures were identified.

Call taker effects were not modeled separately in this study. Instead each call taker/caller pair was considered to be independent of any other call taker/caller pair. Any call taker effect was considered to be dependent on random factors involving the call the call taker happened to answer and the callers and contexts involved in those calls.

### The conventional conversation analysis

#### The personal emergency situation model

**Fig. 1:**
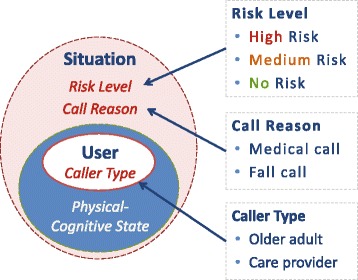
The PES Model characterized by caller type, risk level and call reason

The personal emergency situation model or PES model is one way to characterize a personal emergency situation using three categories: (1) caller type, (2) call reason, and (3) risk level as illustrated in Fig. 1. The PER model is an extension of the PES model and includes a “response type” category that represents the response that was requested or provided to the system user.

This model was developed as part of a separate study looking at the lexical semantics of the words used in the call conversations [81] and is briefly summarized. In an emergency situation, the caller type may be either: (1) the subscriber (for this study only the older adult callers are considered) and (2) a care provider caller. The care provider caller includes both professional paid providers such as nurses or support workers and unpaid, non-professional care providers such as family or friends. Other caller types are possible but will not be considered in this study (e.g., a few calls had two speakers - both older adult and care provider). The call reason describes why a call is made and for this study was determined to be either a: (1) medical or (2) fall call. In this study, a ‘fall call’ was defined as a call where the caller experiences an unintentional fall, is not hurt or hurt minimally, but cannot get up without assistance. Fall calls resulting in physical injury, such as bleeding, were considered to be medical calls. For medical calls, the caller usually needs medical assistance either because of a physical injury, pre-existing medical condition, new illness, or psychological concern. In the PES model, calls were categorized by risk type which includes three basic risk levels: (1) no risk (a false alarm – no assistance needed), (2) medium risk (needs help soon but not a life or death situation), (3) high risk (possible loss of life or limb – a high risk emergency situation) [82]. These risk levels were identified following the “ABC’s of emergency response and calls were later categorized into these risk levels by two individuals, the first author and a physician specializing in geriatric emergency medicine [81].

### The quantitative conversational analysis

The quantitative conversation analysis is divided into four sections. Section I examined whether any patterns exist between the call categories (i.e., caller types, call reasons, risk levels) and the call response types. The call categories significantly related to response type were used as independent factors in the quantitative analyses described in the other three sections. Sections II to IV of the quantitative analysis explored the relationships between various conversational measures and call categories. Section II focused on verbal ability measures, Section III focused on conversational structure measures, and Section IV focused on timing measures.

#### Section I: conversation analysis using call categories

Descriptive statistics were used to describe the calls and to identify caller demographics such as age and gender. Frequency of call categories was also calculated (e.g., how many high risk calls, how many older adult calls). Pearson’s Chi Square statistic and Fisher’s Exact test were used to examine significant relationships between PES model categories and response type.

#### Sections II to IV: analysis of conversational measures

In these three sections various statistical analyses were performed to examine conversational data. Multivariate analysis of variance (MANOVA) tests, univariate analysis of variance (ANOVA) tests, t-tests, and discriminant analyses were used to examine if selected conversational measures could be used to predict call categories.

#### Section II: verbal ability measures and call categories

In this part of the study four aspects of verbal ability were examined: (1) number of words spoken; (2) length of a turn in words; (3) number of one word utterances; and (4) speech disfluency. These measures are commonly used to characterize speech production in conversation [83].

##### Number of caller words spoken

This measure examines how many words each speaker in the conversation utters. The words counted include only the completed words spoken and not words which were partially spoken or interrupted (see ‘mazes’ in the disfluency section further on).

##### Speaker turn length

According to Sacks et al. [84] “the organization of ‘taking turns to talk’ is fundamental to conversation…” (pg.2). In this analysis, a ‘speaker turn’ is defined as the unit of speech or thought communicated by a participant during their turn to talk in a response call conversation. The end of the first speaker’s turn may be signaled either by silence or interruption by the next speaker thereby causing the first speaker to stop speaking. The model of turn-taking is outlined by [84]. Measures of “average turn length (in words)” indicate how many words the caller(s) and call taker utters during their turn to speak. In SALT, the average turn length in words is calculated using all main body words but excludes maze words. A speaker turn length includes all “contiguous utterances of the same speaker” including non-verbal, incomplete, or unintelligible utterances [79]. Utterances were determined phonologically (by content, pausing, intonation changes) as described in the SALT manual [79] and by [85]. In this study examining the length of a speaker turn in words may give some indication as to how the conversation changes depending on the call characteristics.

##### One word utterances

One speaker turn may be composed of one or more speaker utterances either verbal or non-verbal. Examining the number of one word utterances in a call may provide some insight into the frequency of short one word statements used within a call conversation and how this may change depending on different call categories. A simple SDS with limited vocabulary may only be designed to accept one word responses (e.g., ‘yes’ or ‘no’). However, studies suggest that older adults may not always respond to medical questions with simple “yes” or “no” responses [55]. We are interested in knowing how often one word utterances are used to respond to the call taker and if this changes depending on the type of situation.

##### Disfluency

Disfluencies are part of normal speech [86] and may be marked by the presence of mazes. Hall et al. [87] defines a maze as “a marker of linguistic disfluency in spontaneous speech,” p.162. The SALT Help Manual defines a maze as, “any filled pause [e.g., uh, ah], false starts [e.g. and I (ha*) have], repetitions [e.g., (and) and I] and reformulations [e.g. (He and) he said] that are parenthesized in the utterance. … When maze words are removed from the utterance, the remaining words can stand alone.” [79]. Ordinarily, mazes occur when a speaker is expressing an idea that may be abstract, complicated or partially formulated [88]. Research studies suggest that 6–10 % or more of spontaneous speech will contain mazes depending on the discourse and situational context with older adults producing slightly more than younger adults [89–91]. To obtain an estimate of the number of disfluencies above typical expectations, in this study, the proportion of total word mazes occurring more than 10 % of the time in a call is examined. The proportion of total word mazes in SALT is calculated as the number of maze words per total number of words in a conversation.

##### First spoken turn measures

A well-functioning SDS for a smart PERS should be able to adapt its dialogue to the caller and to identify the desired or most appropriate response as quickly as possible. In this analysis, two measures of verbal ability, words per minute and turn length, were examined to see whether certain characteristics of a call could be identified by looking only at the first spoken turn of the caller. Each caller turn may be composed of one or more utterances. The first caller turn is counted as the first turn after the call taker begins the conversation. Caller turns in which the caller does not respond with spoken words are not counted for this analysis.

##### First spoken turn words per minute

An older adult’s overall rate of speech and intelligibility can be affected by physiological changes in the aging body as a result of higher breathing frequency and reduced vocal range, speed and accuracy of structural movement [92]. The measure of ‘first spoken turn words per minute’ was calculated as the number of words, in the caller’s first spoken turn, (including maze words), that occurred after the call taker initiated the conversation. The corresponding ‘words per minute’ rate was obtained by dividing this word count by the time it took for the caller to complete that first spoken turn. The first turn time was measured from the end of the call taker’s previous utterance to the end of the caller’s last utterance in the first spoken turn. Existing literature has previously shown that words per minute for older adults tends to be lower than that of younger individuals [93]. Examining words per minute in this study may provide insight into how verbal fluency differs depending on different call situations focusing on the first caller turn in the call. The question being considered here is whether a SDS might be able to classify a call within the caller’s first spoken turn, based on the speaker’s rate of speech.

##### First spoken turn length in words

The first spoken turn length in words was examined to get a better sense of how many words are typically uttered during this first turn. How many words should a SDS expect to recognize if a user is not constrained to a particular word limit and assuming the computer system asked the same opening question as a live call taker? Are there differences between callers?

#### Section III: conversational structure measures

In this section, three aspects of conversational structure were examined: (1) number of statements, (2) questions, and (3) responses to questions. These measures were selected to examine if any patterns exist in how different calls might be responded to by different callers. Do callers respond differently in PER calls and how do their styles of speaking in a conversation relate to different call categories?

##### Statements, questions, and responses

Research literature on emergency calls [62], Emergency Call Centre protocol [94], and on-site observations of call takers indicate a majority of the queries in the call conversation are by call takers and a majority of the responses to questions in the call conversation are by PERS users. Analyzing the statements, queries, and responses to questions may help identify differences in conversational structure between callers and call takers as well as verify what is expected to occur via the PER provider’s call handling protocol. In SALT, ‘responses to questions’ are defined as any “utterance that immediately follows a question from a different speaker” in the conversation. Statements are utterances that end with a period. Questions are utterances that end with a question mark [77].

#### Section IV: timing measures

In an actual emergency situation, seconds matter. Eight minutes or less is the current recommended target time for 90 % of emergency responses [95–98]. For individuals in cardiac arrest, a response time of 5 min or less has been found to increase survival rates for patients [95, 96, 99]. In a SDS, similar to human call taker, the main goal is to determine what response is required and to initiate an appropriate response as quickly as possible. We define the ‘call taker response time’ as the time between the beginning of the response call conversation to the time when the call taker either ends the conversation (i.e. says “good-bye”) or puts the caller on hold to initiate a call response. Two measures were used to determine response time: (1) number of speaker turns and (2) time in seconds. These two timing measures may prove useful for setting a standard benchmark that the SDS of a smart PERS should meet or exceed. In this study, two call categories: (1) caller type and (2) risk level were used to assess their effect on response time.

##### Number of calls analysed

No risk calls (e.g. false alarms) were not included in this analysis. However, this data is shown on the boxplots where relevant. In Part 1, the conventional conversational analysis, seventy-two (72) of the 84 calls were included in the analysis. 12 calls were excluded including: nine false alarm calls initiated by the older adult subscriber; one follow-up (status update) call by the call taker (e.g., have you been looked after?); and two combination medical calls made by the older adult subscriber and care provider together.

In Part 2, the quantitative conversational analysis, section I and the first turn measures analysis in section II included seventy-two (72) calls. All other analyses in Section II included seventy-one (71) calls. One call outlier was identified and removed because the caller had hearing and communication difficulties resulting in a higher than normal speaker turn number.

## Results

### Part 1: the conventional conversation analysis

#### Response types

The two “response type” categories identified from the call conversations included: (1) EMS and (2) other non-EMS responders, as previously defined in the “data collection” section. An “all responder” category could also be considered representing the scenario where “all responders” (i.e., EMS and non-EMS) are called to attend a personal emergency situation.

#### Ambulance vs. Paramedic

In reading through the response call transcripts, one call in particular revealed perceived differences between the target response terms “ambulance” and “paramedic.” “Ambulance” is a term used to describe the emergency vehicle used to transport a patient from home to a healthcare facility. “Paramedic” is the term used to describe the medical care personnel who would drive or travel in the ambulance and who would usually be the first responder to an emergency scene. This finding is interesting because the caller was not using the terms interchangeably even though paramedical personnel drive the ambulance vehicles to attend emergency situations. The caller specifically declined the proposal for an “ambulance” and requested a “paramedic” be sent instead. There may be many possible reasons for the caller not wanting the ambulance including not wanting to leave the home unattended, fear of going to the hospital, not wanting the ambulance cost. The following excerpt from the transcript (call example 1) is presented below (CT = call taker, C = caller (older adult), arrow brackets < > mark overlapping speech, parentheses () mark repeated speech or mazes, and curly parentheses {} mark comments or other noises):

Call Example 1:

**Table:** 

Line 1	*CT: Do you need an ambulance?*
Line 2	*C: {Grunt} No, I don’t need an ambulance, I thought paramedics or something <> to check me over.*
Line 3	*CT: <Yes>, you want the paramedics to come and check you over?*
Line 4	*C: Yeah, (I) I don’t want (an) an ambulance <>.*
Line 5	*CT: <Oh > .*
Line 6	*C: <Cause > I’m not going anywhere.*

In situations where callers are requesting non-EMS responders, it is important to note that even when medical attention may be necessary, the PERS user may want someone other than EMS support. In call example 2, the older adult is feeling weak and vomiting. However, when asked if an ambulance is required, this caller requests the daughter as the preferred response.

Call Example 2:

**Table:** 

Line 1	*CT: Hello, how are you?*
Line 2	*C: Oh, I need help (weak, shaky voice).*
Line 3	*CT: What’s wrong?*
Line 4	*C: (Oh I) I keep throwing up and going to the bathroom.*
Line 5	*CT: (You) You’re vomiting?*
Line 6	*CT: How long has this been going on?*
Line 7	*C: Oh, it just started now < xx>. {xx = two unintelligible words}*
Line 8	*CT: <Okay > .*
Line 9	*CT: Okay, is there anyone there with you right now?*
Line 10	*C: No.*
Line 11	*CT: Okay.*
Line 12	CT: Okay so do you want me to call an ambulance for you or did < you wan*>>
Line 13	*{CT was cut-off mid-word}*
Line 14	*C: <No > no, I just want you to call my daughter.*

Call Example 2 shows how the call taker quickly assesses the situation and makes an initial decision about what response to provide. From lines 4 to 9, the call taker identifies the problem and if anyone is onsite. At line 12, the call taker suggests an ambulance. At line 13, the older adult asks for her daughter.

In call example 3, the older adult would like assistance and the paramedics are offered, but the preference is for someone else. Unfortunately, there are no other responders on this caller’s list. The call operator concludes that only paramedics can be sent in this situation.

Call Example 3:

**Table:** 

Line 1	C: (Eh) I wonder if you could (have s*) send somebody down to my place?
Line 2	*CT: And who would you like me to call for you?*
Line 3	*C: (Eh) well x {possible grunt} nobody.*
Line 4	*CT: Would you like the paramedics?*
Line 5	*C: (Ah) can you get somebody else?*
Line 6	*CT: Somebody else, other than the paramedics?*
Line 7	*C: That’s right.*
Line 8	*CT: Oh well (uh) you don’t have any responders on your file.*
Line 9	*CT: (Uh), is there anyone in particular you would like me to call?*
Line 10	*C: No.*
Line 11	*CT: Okay, we can only call the paramedics.*

In Call Example 3, the call taker is asked for assistance in line 1 right away. In line 2, the call taker wants the caller to identify a specific desired responder. However, from lines 3–10, the call taker discovers that even though the caller does not want EMS, this is the only response that can be provided. In line 11, she explains this to the caller.

These excerpts show the importance placed on having different types of target responses and also demonstrates how actual PER call dialogue may be challenging for a SDS to handle. For example, in Call Example 1, line 2, the caller negatively responds to the call taker’s responder type proposal and immediately suggests her desired response. A “grunt” at the beginning of the call would be considered a word by an ASR system which might be difficult to interpret. Call Example 3 also shows a special situation where even when help is offered and refused, there may need to be a dialogue state that deals with the situation where only one response is possible.

Guided by the call conversations, the final response type categories selected included: (1) ambulance, (2) paramedic, (3) other responder, and (4) all responders (EMS and other).

#### The personal emergency response (PER) model

**Fig. 2:**
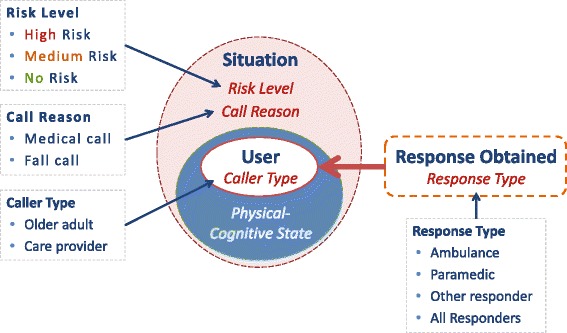
The PER model

Figure 2 illustrates a model of the PER event classified by caller type, risk level, call reason, and response type. Sub-categories for each classification are shown.

### Part 2: the quantitative conversational analysis

#### Section I: conversation analysis using call categories

##### Demographics of personal emergency response call callers

Fifty (50) calls were made by older adult callers and 22 were made by care providers. Subscriber age at the time of the call was determined for 53 of 84 calls (63 %). Mean age was 82 years (standard deviation (StdDev) = 8.79) with the youngest known age being 51 years and the oldest known age being 100 years old. There were 69 female and 15 males subscribers, with gender being inferred from the conversation (i.e. use of “he” or “she” by the other caller) or by voice pitch (low for males, higher for females). The higher female caller ratio observed in the collection of response calls is common amongst PERS users and this age group [11, 12, 17, 100].

##### PES model classifications and response type associations

**Fig. 3:**
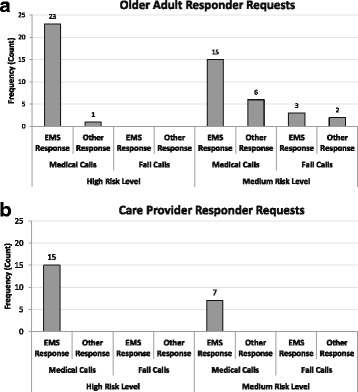
Caller Responses broken down by risk level and call reason

In this analysis, associations between caller type, risk level, call reason, and response type were examined. The frequency of calls broken down by caller type (older adult vs. care provider), risk level (high vs. medium), call reason (fall vs. medical), and response type (EMS vs. Other Responders) are shown in Figs. 3a (older adult response) and 3*b (care provider responses)*.

Pearson’s Chi Square statistic and Fisher’s Exact test (for cases where counts are less than five), revealed no significant associations between caller type and call reason; caller type and risk level; and call reason and response type. The three significant associations are summarized next:

1. Caller Type vs. Response Type
  A borderline significant relationship was found using Fisher’s Exact test between caller type and response type, *p* = 0.049 (Exact sig., 2-sided) suggesting that a difference exists between the response-type requested by different callers. Specifically, older adult and care provider callers both made requests for EMS responses, however, older adults also made requests for other responders.
2. Risk Level vs. Call Reason
  A significant relationship was found using Fisher’s Exact test between risk level and call reason, *p* = 0.017 (Exact sig., 2-sided) suggesting that high risk calls were more likely to be medically related than fall related.
3. Risk Level vs. Response Type
  A significant relationship was found using Fisher’s Exact test between risk level and response type, *p* = 0.009 (Exact sig., 2-sided) suggesting that high risk calls were more likely to lead to an EMS response whereas medium risk calls could result in requests for other response types.

##### Breakdown of response types

With respect to response type, care providers requested EMS responses 100 % of the time for both high and medium risk medical situations. Of the three calls requesting a ‘paramedic’ response, two calls were high risk and one was medium risk. For older adult callers, EMS responses were requested 96 % of the time in high risk, medical call situations: 19 out of 24 calls were for ambulances. The other calls consisted of two calls for the ‘paramedics’, one call for an ‘other responder’, and two calls for both ‘EMS and other’ responders. In medium risk medical call situations, EMS requests dropped to 71 % with 12 out of 21 calls for the ‘ambulance’, two calls for the ‘paramedics’, six calls for ‘other responders’, and one call for ‘EMS and other’ responders. Medium risk fall situations (five calls total) exhibited a fairly distributed range of requests with two calls for the ambulance, one call for the paramedic, and two calls for ‘other responders’.

Significant group relationships between caller type and response type, risk level and call reason, and risk level and response type suggest that the desired ‘response type’ may be predicted to some degree if the ‘caller type’ and/or ‘risk level’ of a call can be determined. Identifying the ‘call reason’ may also help in identifying ‘risk level’ or vice versa which could then be used to estimate “response type”. Due to the small number of fall calls identified based on the fall definition used, the subsequent conversational analyses focused on classifying calls based on two call characteristics: caller type and risk level.

#### Sections II to IV: conversation analysis using conversational measures

The analyses performed took into account unbalanced group counts. Where relevant, univariate analysis of variance (ANOVA) tests and t-tests were conducted following MANOVA tests to compare different groups with significant multivariate effects. Discriminant analyses were also conducted where relevant to examine which and how well certain measures could be used to predict significant independent factors.

#### Section II: verbal ability measures and call categories

Seventy one calls were used for this analysis. Six verbal ability measures were examined in this section: (1) number of complete words (NumWds); (2) average turn length in words (AvgTnLgth); (3) number of one word utterances (OneWrdUtts); (4) proportion of total words with mazes (PrctMazes); (5) first turn words spoken per minute (1stTnWPM); and (6) first turn length in words (1stTnLgth).

To begin the analyses, NumWds, AvgTnLgth, and OneWrdUtts measures were analyzed together using a mixed MANOVA and three independent factors: (1) caller type (older adult and care provider); (2) risk level (high and medium); and (3) speaker type (callers and call takers). Speaker type is an independent factor used to differentiate between the PERS callers and the call takers. The call taker group is thus used as a ‘comparison group of convenience’. Speaker type was a ‘within subjects factor’ and risk level and caller type were ‘between subject factors’. The PrctMazes measure was examined independently because the data could not be normalized sufficiently to include in the mixed MANOVA and Pearson’s correlation coefficient with the other measures was below 0.3. To get a better sense of whether the caller type or risk level could be determined based on the caller’s first spoken utterance, 1stTnWPM and 1stTnLgth measures were analyzed together using a two-way MANOVA with the two independent factors: (1) caller type (older adult and care provider), and (2) risk level (high and medium).

Prior to applying statistical analyses transformations were required to normalize some measures as outlined in [101]. Log10(x) transformations were applied to the NumWds and AvgTnLgth data; a Log10(x + 1) transformation was applied to the OneWrdUtts data; a square root transformation was applied to the PrctMazes data; and a ln transformation was applied to 1stTnLgth. No transformation was required for 1stTnWPM as the data was normally distributed. Measures analyzed in the same MANOVA were all moderately correlated with Pearson’s correlation coefficients ranging from 0.3 to 0.7, *p* < 0.001.

##### Analysis 1: NumWds, AvgTnLgth, and OneWrdUtts with three independent factors

**Fig. 4:**
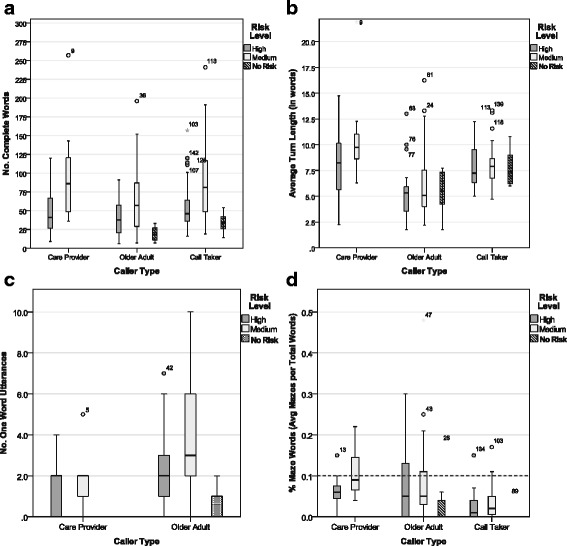
Verbal ability measures broken down by risk level and speaker type

The results of the mixed MANOVA revealed significant within subjects multivariate effects for *speaker type*, Wilks’ λ = 0.784, F(3,65) = 5.98, *p* = 0.001, η2 = 0.216, power = 0.946, and for the interaction between *speaker and caller type*, Wilks’ λ = 0.748, F(3,65) = 7.30, *p* < 0.001, η2 = 0.252, power = 0.979. There was no significant multivariate interaction effect between speaker type and risk level nor for the three-way interactions between speaker type, caller type and risk level. Between subjects, significant multivariate effects were obtained for both *caller type*, Wilks’ λ = 0.849, F(3,65) = 3.86, *p* < 0.05, η^2^ = 0.151, power = 0.8, and *risk level*, Wilks’ λ = 0.866, F(3,65) = 3.35, *p* < 0.05, η^2^ = 0.134, power = 0.735. There was no significant multivariate interaction effect on caller type and risk level. In Fig. 4, box plots can be found illustrating the verbal ability measures (a) NumWds; (b) AvgTnLgth; and (c) OneWrdUtts by caller type and speaker type, and broken down by risk level.

##### Number of complete words

Boxplots illustrating NumWds can be found in Fig. 4a. Caller takers were found to use significantly more words compared to callers, F(1,67) = 7.88, *p* < 0.05,,η^2^ = 0.105, power = 0.790 (univariate test for *speaker type*). There was no significant effect for caller type. A significant interaction effect was obtained between *speaker type and caller type*, F(1,67) = 21.40, *p* < 0.001, η^2^ = 0.242, power = 0.995 and between *speaker type and risk level*, F(1,67) = 4.84, *p* < 0.05, η^2^ = 0.067, power = 0.582. Paired t-tests conducted at each caller level between callers and call takers revealed significant differences in the NumWrds used between *call takers* (Mean = 70.18, StdDev 43.47) and *older adult callers* (Mean = 54.61, StdDev 41.37), t(48) = −6.7, *p* < 0.001,but no significance difference between call takers and care provider callers (Mean = 65.45, StdDev 54.28). These results suggest that call takers spoke significantly more words than the older adult callers, but care providers and older adults spoke a similar number of words.

The NumWrds spoken also differed between high (Mean =49.26, StdDev = 28.91) and medium (Mean = 82.14, StdDev = 53.60) risk levels regardless of caller or speaker type, F(1,67) = 8.79, *p* < 0.05, η^2^ = 0.116, power = 0.832 (univariate test for *risk level*). No significant interaction effects were obtained. Independent samples t-tests conducted for each caller level and the call taker group between risk levels revealed significant differences between *high and medium risk levels* for *older adult callers*, t(47) = −2.35, *p* = 0.023, care providers, t(20) = −2.34, *p* = 0.030, and *call takers*, t(69) = −3.24, *p* = 0.002. These results suggest that all speakers, older adults, care providers, and call takers, spoke significantly fewer NumWrds during high risk calls than medium risk calls.

##### Average turn length in words

Boxplots illustrating AvgTnLgth can be found in Fig. 4b *. Care provider* callers were found to have significantly longer AvgTnLgth compared to *older adult callers*, F(1,67) = 8.45, *p* < 0.01, η^2^ = 0.112, power = 0.818 (univariate test for *caller type*); and a borderline significant mean difference was also found between the *caller types* and *call takers*, F(1,67) = 3.86, *p* = 0.054, η^2^ = 0.054, power = 0.491 (univariate test for *speaker type*). A significant interaction effect was obtained between *speaker type and caller type*, F(1,67) = 22.03, *p* < 0.001, η^2^ = 0.247, power = 0.996. Paired t-tests conducted at each caller type level between callers and call takers revealed no significant difference in AvgTnLgth between care provider callers (Mean = 9.13, StdDev 4.2) and call takers (Mean = 7.88, StdDev 1.95) but a significant difference in AvgTnLgth between *older adult callers* (Mean = 5.81, StdDev 3.14) and *call takers*, t(48) = −6.12, *p* < 0.001. These results suggest that care provider callers have AvgTnLgth comparable to call takers, but older adults tend to have significantly shorter AvgTnLgth compared to both call takers and care providers. AvgTnLgth was not found to be significantly different between risk levels and no significant effects were obtained between speaker type and risk level or between caller type and risk level.

##### Number of one word utterances

Boxplots for OneWrdUtts can be found in Fig. 4c. *Care providers* had borderline significantly fewer OneWrdUtts than *older adult callers*, F(1,67) = 3.93, *p* = 0.052, η^2^ = 0.055, power = 0.497 (univariate test for *caller type*); and the *caller group* differed significantly from that of the *call taker group*, F(1,67) = 6.78, *p* < 0.05, η^2^ = 0.092, power = 0.728 (univariate tests *speaker type*). Paired samples t-tests conducted between each caller level and the associated call takers revealed a significant difference in OneWrdUtts between *older adults callers* (Mean = 3.06, StdDev 2.49) and *call takers* (Mean = 1.54, StdDev 1.4), t(48) = 4.23, *p* < 0.001, but no significant difference between care providers callers (Mean = 1.64, StdDev 1.40) and call takers. These findings suggest that older adult callers made significantly more one word utterances than both care provider callers and call takers, while one word utterances are similar between care provider callers and call takers.

The OneWrdUtts spoken also differed between high and medium risk levels regardless of caller or speaker type, F(1,67) = 6.23, *p* = 0.015, η^2^ = 0.085, power = 0.692 (univariate test for *risk level*). No significant interaction effects were obtained. Independent samples t-tests conducted for each caller level and the call taker group between risk levels revealed significant differences between *high and medium risk levels* for *older adult callers*, t(47) = −2.27, *p* = 0.028, and *call takers*, t(69) = −2.70, *p* = 0.009, but no significant difference was observed for the care provider callers. These results suggest that both older adult callers and call takers made significantly fewer OneWrdUtts during high risk calls than medium risk calls, while care providers make approximately the same number of OneWrdUtts across risk levels.

##### Discriminant analysis

A discriminant analysis was used to examine the predictability of caller type using three predictor variables: NumWrds, AvgTnLgth, and OneWrdUtts. Box’s M test was not significant at the 0.05 level. The discriminant function revealed a significant association between caller type and all predictors. Entering independent variables together, Wilks λ = 0.774, χ^2^(3) = 17.306, canonical correlation = 0.476, *p* = 0.001. 22.6 % of the variance between older adult and care provider speakers was accounted for. Using the standardized canonical discriminant function coefficients, the discriminant function revealed two major predictors: *NumWrds* and *AvgTnLgth*; discriminant function = (−0.771 × NumWrds) + (−0.027 × OneWrdUtts) + (1.387 × AvgTnLgth). Classification based on the discriminant function and group centroids (Care Provider = 0.795; Older Adult = −0.357) using the original group cases resulted in moderate success at 77.5 % of cases being correctly classified. 87.8 % of older adults and 54.5 % of care providers (out of 22 Care Provider and 49 Older Adult cases). Using cross-validated classification the number of correctly classified cases dropped slightly to 73.2 %, with the older adult percentage of correctly classified cases dropping to 85.7 % and the care provider percentage of correctly classified cases dropping to 45.5 %. These classification results apply only to the cases used in this study.

Looking at risk level predictability (i.e., high and medium risk levels) using the same three predictors, the discriminant function revealed a significant association between risk levels and all predictors. Entering independent variables together, Wilks λ = 0.845, χ^2^(3) = 11.362, canonical correlation = 0.394, *p* = 0.010. Box’s M test was not significant at the 0.05 level. 15.5 % of the variance between high and no risk levels was accounted for. Using the standardized canonical discriminant function coefficients, the discriminant function revealed one major predictor: *NumWrds*; discriminant function = (1.003 × NumWrds) + (0.368 × OneWrdUtts) + (−0.352 × AvgTnLgth). Classification based on the discriminant function and group centroids (high risk = −.382; medium risk = 0.466) using the original group cases resulted in moderate success at 67.6 % of cases being correctly classified, 76.9 % at the high risk level and 56.3 % at the medium risk level (out of 39 high risk and 32 medium risk cases). The results using cross-validated classification dropped the number of correctly classified cases slightly to 63.4 % with only the high risk level percentage of correctly classified cases dropping to 69.2 %. These classification results apply only to the cases used in this study.

##### Analysis 2: percent maze words and three independent factors

The PrctMazes was examined using paired t-tests for caller type, risk level and speaker type. Boxplots for PrctMazes can be found in Fig. 4d. The PrctMazes spoken by the callers collectively was higher than for call takers. Paired t-tests were conducted to compare the PrctMazes between caller types (i.e. care provider and older adult) and between speaker types (i.e. callers and call takers). No significant difference was observed between care provider and older adult callers, but *call takers* spoke significantly lower PrctMazes compared to the *callers* combined, t(70) = 5.35, *p* < 0.001. Independent t-tests were conducted to compare the PrctMazes between risk levels, and for each caller and speaker types at different risk levels. No significant difference was observed between the overall risk levels or between the older adult and call taker groups at the different risk levels. A borderline significant result was obtained for the *care provider group* at different *risk levels*, t(20) = −2.03, *p* = 0.056. These results show an increase in PrctMazes produced by the care provider during medium risk calls but it is borderline significant.

The frequency of mazes occurring more than 10 % (or 0.1) of the total words, (see dotted line in Fig. 4d), was also calculated for each speaker. Older adults expressed a greater number of mazes per total number of words occurring more than 10 % of the time, 34.7 % of transcripts (17 times out of 49 calls), compared to care provider callers, 22.7 % of transcripts (5 of 22 calls), and call takers, 5.6 % of transcripts (4 of 71 calls). Using the Chi-Square test, frequencies between older adult and care provider callers were not found to be significantly different, however, when call taker frequencies were included a significant difference was obtained, χ^2^(2) = 16.71, *p* < 0.001.

##### Analysis 3: 1stTnLgth and 1stTnWPM with two independent factors

**Fig. 5:**
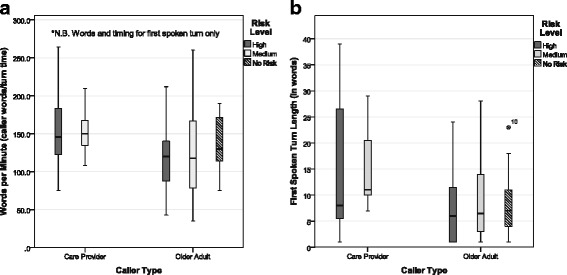
First spoken turn measures broken down by caller type and risk level

Seventy two calls were used for this analysis. The results of the two-way MANOVA revealed significant multivariate main effects for *caller typ*e, Wilks’ λ = 0.892, F(2,67) = 4.04, *p* < 0.05, η^2^ = 0.108, power = 0.702. There was no significant multivariate main effect for risk level or for the interaction between caller type and risk level. In Fig. 5, two box plots illustrate measures (a) 1stTn WPM and (b) 1stTnLgth, broken down by caller type and risk level.

##### First spoken turn words per minute

Boxplots for 1stTnWPM can be found in Fig. 5a. The 1stTnWPM spoken by *older adult callers* (Mean = 122.57, StdDev = 52.93) was significantly lower than that of *care provider callers* (Mean = 156.03, StdDev = 48.98), F(1,68) = 5.46, *p* = 0.22, η^2^ = 0.074, power =0.634 (univariate test for caller type). These results suggest that older adult callers speak significantly fewer WPM in the first spoken turn of the call conversation compared to the care provider callers.

##### First turn length in words

Boxplots of 1stTnLgnth can be found in Fig. 5b. *Care provider callers* (Mean = 15.32, StdDev = 11.38) were found to have significantly longer 1stTnLgths compared to *older adult callers* (Mean = 8.22, StdDev = 7.14), F(1,68) = 7.65, *p* < 0.01, η^2^ = 0.101, power = 0.778 (univariate test for caller type). These results suggest that older adults tend to have significantly shorter first turn lengths in words compared to care providers.

##### Discriminant analysis

A discriminant analysis was used to examine speaker predictability between caller type (older adult and care provider) using two predictor variables: 1stTnWPM and 1stTnLgth. Box’s M was non-significant at the 0.05 level. The discriminant function revealed a significant association between caller type and all predictors. Entering independent variables together, Wilks λ = 0.895, χ^2^(3) = 7.514, canonical correlation = 0.323, *p* = 0.023. 10.5 % of the variance between older adult and care provider speakers could be accounted for by the predictor variables. Using the standardized canonical discriminant function coefficients, the discriminant function revealed two predictors; 1stTnLgth and 1stTnWPM; discriminant function = (0.610 × 1stTnLgth) + (0.494 × 1stTnWPM). Classification based on the discriminant function and group centroids (Care Provider = 0.503; Older Adult = −0.226) using the original group cases resulted in moderate success at 73.2 % of original cases being correctly classified, 95.9 % of older adults but only 22.7 % of care providers (out of 22 Care Provider and 50 Older Adult cases). Classification using cross validation in SPSS is performed where each case is classified by the functions derived from all cases except for the case of interest. The results using cross-validated classification dropped the number of correctly classified cases to 67.6 % with the older adult and care provider percentage of correctly classified cases dropping to 91.8 % and 13.6 % respectively. These classification results apply only to the cases used in this study.

#### Section III: analysis of conversational structure measures

71 calls were used for this analysis. Three conversational structure measures were examined in this section: (1) number of statements (NumStmt); (2) number of questions (NumQues); and (3) number of responses to questions (NumResQues). NumStmt, NumQues, and NumResQues were analyzed together using a mixed MANOVA and three independent factors: (1) caller type (older adult and care provider), (2) risk level (high and medium), and (3) speaker type (callers and call takers). As in Section II, Speaker type was included in this analysis to differentiate between PERS callers and the ‘call taker’ group. The call taker group was used as a ‘comparison group of convenience. ‘Speaker type was a ‘within subjects factor’ and risk level and caller type were the ‘between subject factors’. Prior to applying statistical analyses some transformations were required in order to normalize the data as outlined in [101]. Log10(x + 1) transformations were applied to NumStmt, NumQues, and NumResQues. Measures analyzed in the MANOVA were all moderately correlated with Pearson’s correlation coefficients ranging from 0.3 to 0.7, *p* < 0.001.

**Fig. 6:**
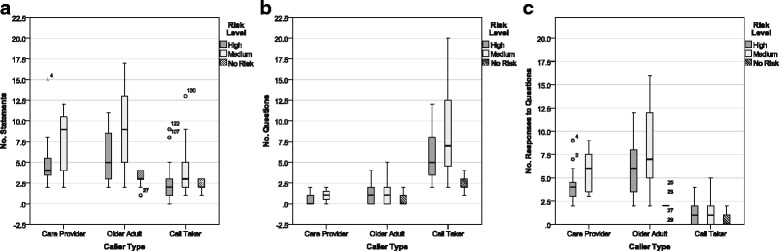
Conversational measures broken down by risk level for caller and speaker types

The results of the mixed MANOVA revealed a significant within subjects multivariate effect for *speaker type*, Wilks’ λ = 0.192, F(3,65) = 91.44, *p* < 0.001, η^2^ = 0.808, power = 1.0. A significant between subjects multivariate effect was obtained for *caller type*, Wilks’ λ = 0.861, F(3,65) = 3.485, *p* < 0.05, η^2^ = 0.139, power = 0.754, and a significant multivariate effect was obtained for *risk level*, Wilks’ λ = 0.871, F(3,65) = 3.20, *p* < 0.05, η^2^ = 0.129, power = 0.714. All 2 and 3 way interaction effects were non-significant. In Fig. 6, three box plots illustrate the conversational structure measures (a) mean NumStmt; (b) mean NumQues; and (c) mean NumResQues, broken down by risk level and caller and speaker types.

##### Number of statements

Box plots of NumStmt can be found in Fig. 6a *.* The NumStmt spoken by callers is similar between older adult and care provider callers (no significant effects for caller type); but differ as a combined *caller group* from that of the *call taker group*, F(1,67) = 125.01, *p* < 0.001, η^2^ = 0.651, power = 1.0 (univariate test for *speaker type*). Paired samples t-tests conducted at each caller level between callers and call takers revealed significant differences in NumStmt between care providers and call takers, t(21) = 8.43, *p* < 0.001, and older adults and call takers, t(48) = 10.43, *p* < 0.001. These findings show that both callers made significantly more statements than the call takers during the response call.

The NumStmt spoken at *high risk levels* was found to differ significantly from those at *medium risk levels*, F(1,67) = 8.82, *p* < 0.01, η^2^ = 0.116, power = 0.833 (univariate test for risk level). No significant interaction effects were obtained between caller type, speaker type and/or risk level. Independent samples t-tests conducted for each caller level and the call taker group between risk levels revealed significant differences between *high and medium risk levels* for *older adult callers*, t(47) = −2.82, *p* = 0.007, and *call takers*, t(69) = −3.34, *p* = 0.001, but no significant difference was observed for the care provider callers. These results suggest that both older adult callers and call takers make significantly fewer statements during high risk calls than medium risk calls, however care providers make approximately the same NumStmt across risk levels.

##### Number of questions

Boxplots of NumQues can be found in Fig. 6b. The NumQues asked by *care provider callers* was significantly less than *older adult callers*, F(1,67) = 7.31, *p* < 0.01, η^2^ = 0.098, power = 0.759 (univariate test for *caller type*); and the *caller group* NumQues differed significantly from that of the *call taker group*, F(1,67) = 269.46, *p* < 0.001, η^2^ = 0.801, power = 1.0 (univariate test for *speaker type*). Paired samples t-tests conducted between each caller level and the associated call takers revealed significant differences in NumQues between care providers and call takers, t(21) = −12.42, *p* < 0.001, and older adults and call takers, t(48) = −15.20, *p* < 0.001. These findings suggest that both callers asked significantly less questions than the call takers during the response calls and care providers asked less questions than older adult callers. There was no significant effect for risk level and no significant interaction effects were obtained.

##### Number of responses to questions

Boxplots of NumResQues can be found in Fig. 6c *.* The *care provider callers* had significantly less NumResQues than *older adult callers*, F(1,67) = 5.35, *p* < 0.05, η^2^ = 0.074, power = 0.625 (univariate test for *caller type*); and the *caller group* had significantly more NumResQues than the *call taker group*, F(1,67) = 267.50, *p* < 0.001, η^2^ = 0.800, power = 1.0 (univariate test for speaker type). Paired samples t-tests conducted between each caller level and the associated call takers revealed significant differences in NumResQues between *care providers* and *call takers*, t(21) = 12.00, *p* < 0.001, and *older adults and call takers*, t(48) = 15.06, *p* < 0.001. These findings confirmed that both care provider and older adult callers responded to significantly more questions than call takers, and older adults responded to more questions than the care provider. The NumResQues spoken did not differ significantly across risk levels. No significant interaction effects were obtained.

#### Section IV: analysis of timing measures

**Fig. 7:**
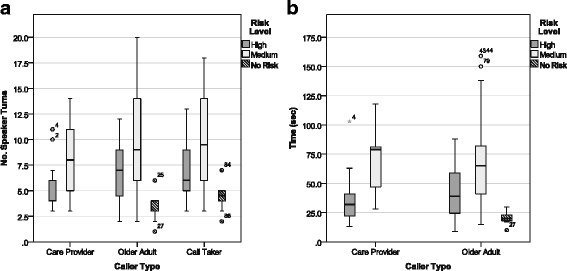
Timing measures broken down by risk level for caller and speaker types

Timing measures included: number of speaker turns (NumSpkrTns) and time in seconds.(TimeSec) Log10(x) transformations were applied to these timing measures to normalize the data. A high and significant Pearson’s correlation coefficient of 0.8, *p* < 0.001, was observed between NumSpkrTns and TimeSec. As a result, the TimeSec measure was not included with NumSpkrTns in the analysis. A mixed ANOVA was conducted on the NumSpkrTns data and examined separately. In Fig. 7, two box plots illustrate the measures (*A*) mean NumSpkrTns and (*B*) mean TimeSec, broken down by caller type and risk level.

##### Number of speaker turns

Boxplots of NumSpkrTns can be found in Fig. 7a. Significant differences were found in NumSpkrTrns between *callers* (Mean = 7.85, StdDev = 4.24) and *call takers* (Mean = 9.27, StdDev = 4.6). Mixed ANOVA results revealed a significant within subjects multivariate effect for *speaker type*, Wilks’ λ = 0.599, F(1,67) = 44.82, *p* < 0.001, η^2^ = 0.401, power = 1.0. The difference in NumSpkrTns between *care provider callers* (Mean = 6.27, StdDev = 3.15) *and older adult callers* (Mean = 8.55, StdDev = 4.5) was not found to be statistically significant. Paired samples t-tests conducted between each caller level and the associated call takers revealed a significant difference between *care provider callers* and *call takers*, t(21) = −5.17, *p* < 0.001, and between *older adult callers* and *call takers*, t(48) = −5.70, *p* < 0.001. These results suggest that older adult and care provider callers speak on average fewer NumSpkrTns than call takers. This finding coincides with the fact that the call takers are managing the conversation and usually are the first and last to speak.

*High risk calls* (Mean = 6.28, StdDev = 2.79) were found to require fewer NumSpkrTns than *medium risk calls* (Mean = 9.75, StdDev 4.93). Mixed ANOVA results revealed, a significant between subjects univariate effect for *risk level*, F(1,67) = 7.61, *p* = 0.007, η^2^ = 0.102, power = 0.776 but no significant differences were observed for caller type nor for any 2 or 3 way interactions within or between subjects. Box’s M and Levene’s Tests were all non-significant at the 0.05 level. No risk calls had a mean of 3.50 NumSpkrTns with a StdDev = 1.35. Independent samples t-tests conducted for each caller level and the call taker group between risk levels revealed significant differences between *high and medium risk levels* for *older adult callers*, t(47) = −2.33, *p* = 0.024, and *call takers*, t(69) = −3.34, *p* = 0.001, but no significant difference was observed for the care provider callers (close at t(20) = −1.82, *p* = 0.084). These results suggest that both older adult callers and call takers take significantly fewer NumSpkrTns during high risk calls, while care provider callers require approximately the same NumSpkrTns across risk levels.

##### Time in seconds

Collectively, the total time of the 71 calls combined was 67 mins (4019 s). The results of a two-way ANOVA examining the relationship between call taker’s response time ‘time in seconds’ with caller type (care provider and older adult groups) and risk level (high and medium risk levels) revealed a significant difference for *risk level*, F(1, 67) = 13.31, *p* = 0.001, but no significant difference for caller type nor the interaction between caller type and risk level. These results suggest that high risk calls (Mean = 40.64 s, StdDev 22.25) have a lower response time than medium risk calls (Mean = 69.59 s, StdDev 39.16) as observed in Fig. 7b. The average response time for no risk calls is 20.70 s, StdDev 1.73.

## Discussion

### Part 1: qualitative conversation analysis of calls

#### The PER model

The categories of PER model features were determined qualitatively from re-occurring themes identified from the transcripts of real PER calls.

#### PERS users

Care providers were identified as PERS users or callers in 22 of the 72 calls used in this study (30.6 %). So, the question arises as to why care providers would use the PERS as opposed to dialing 911 directly using the telephone? Perhaps pushing the PERS button is easier or quicker and would allow them to keep their hands free to actively care for the older adult while making the call. Conversely, pushing the button might actually slow down the process of obtaining emergency assistance because the caller would first have to go through the call taker before reaching EMS. During an on-site visit to an EMS call centre, it was mentioned by an EMS call taker that care providers may be instructed to use the PERS button instead of the phone so that the PER service provider can keep track of their client’s emergency events. In Canada, if an ambulance transfers a patient from home to hospital, this cost is typically born by the person transported. Another possible reason why care providers may use the PERS could be to shift the responsibility of initiating the emergency call to the call taker. Future studies may want to consider questioning the care provider why they push the PERS button.

#### Call reason & risk level

In terms of call reason, many more medical calls were made compared to fall calls. The greater number of medical calls may be due, in part, to the definition used for fall calls in the study, “unintentional falls not resulting in injury.” This definition essentially excluded fall calls from the ‘high’ (emergency) risk level category and only medium risk level fall calls made by older adult callers were identified. The main reason for using this definition for a fall was because caller health information was limited to what could be obtained from the call itself. It was difficult to determine in some calls whether a fall with resulting injury was caused by an underlying medical condition or purely accidental (e.g., person tripped). With the definition used, falls without physical injury could be isolated and examined to see if these calls elicited different responses from PERS users. Based on this definition, fall calls were not observed for care providers in medium risk situations. Presumably, care providers would only need to seek extra assistance if they were not able to help the older adult get up on their own (e.g., patient too heavy or is injured, care provider too weak). Future studies may wish to consider alternate definitions for a fall. For example, a call might be classified as a fall call if the caller mentions that they fell, regardless of whether an injury resulted from the fall or not.

#### Response type & risk level

Looking at the data purely in terms of numbers, for the call response type, because care provider callers were found to request EMS services 100 % of the time, it would seem pertinent for the smart PERS to offer EMS services as a first response option to care provider callers. For older adult callers in high risk situations, an EMS suggestion also seems to be appropriate. However in medium risk situations, an EMS response might work for approximately 70 % of the medical calls and 50 % of the fall calls. The lower number of EMS requests in fall calls may be a result of the fall definition. If the person is not injured and just needs assistance getting up, it makes sense that an ambulance is not always requested. For medium risk level situations, if the older adult caller does not specify up front who they want called and “fall” related words are not mentioned, perhaps suggesting EMS would still be best as a default option. The additional length of time required to suggest an EMS response and wait for a response may make up one additional speaker turn at a minimum.

Looking at the conversation as opposed to the numbers, older adult callers may be sensitive to the different latent meaning behind the words “ambulance” versus “paramedic.” One possible difference in meaning involves the designation of “ambulance” as a vehicle that will take the individual away to be cared for in the hospital versus the designation of “paramedics” as the emergency medical care professionals who will come to the home to check on him/her and see how assistance can be provided. This “fear in leaving the home” may arise for various reasons, for example, fear of losing independence in a hospital or fear of leaving behind a pet or plants. In situations where the older adult is trying to maintain his/her independence, these differences in terms may be very significant. With respect to the smart PERS technology design, using the term “paramedic” to offer assistance may be seen as less aggressive than the term “ambulance,” especially in medium risk level calls made by older adults. The term “ambulance” may be perfectly fine to use in high risk situations when the caller clearly wants to go to the hospital (e.g., makes a request for hospital or ambulance) or can only receive medical care in the hospital (e.g., stroke, heart attack).

The call examples where the caller declines an EMS service and instead requests a non-EMS responder may further suggest that in fact defaulting to the EMS service may not be the best option for certain situations. In medium risk situations, it may be best for the smart PERS to offer the caller the choice between speaking to a call taker or a non-EMS responder, with the default being a first responder previously selected by the older adult subscriber. In the case where no first responder is listed, the default would be the call taker. The offer for an “ambulance” might only then appear first if the smart PERS classifies a call as one of high risk. For example, the person may not be moving (as seen through the system video camera), may not respond verbally, or has mentioned possible high risk terms such as ‘stroke’, ‘heart attack’, ‘need oxygen’, or ‘can’t breathe’. Future studies might consider examining these options in the smart PERS response dialogue with target users. For example, the study might consider what responses the PERS should offer (e.g., ambulance or call taker), when the responses should be offered in the dialogue (e.g., as default, immediately, after response call classification?), and how target responses should be offered (e.g., what words should be used?).

### Part 2: quantitative conversational analysis of calls

The results of the analyses in part 2 of the study suggest that older adult callers use different conversational strategies or engage in different types of dialogue from care providers when conversing with the call taker. Significant differences in the way the care provider responds versus the way the older adult caller responds were observed.

#### Measures using the entire call conversation

Older adults, care providers, and call takers all seem to use, on average, a similar number of words (excluding mazes and incomplete words) over the course of the call conversation. This word count number drops when moving from medium to high risk situations and coincides with the fact that the high risk calls are generally shorter in length. In high risk situations, it seems that less needs to be said by everyone before the call taker initiates a call response.

Taking a closer look at the length of turns in conversation, older adult callers had on average shorter turn lengths (in words) and used more one word responses than care providers. In contrast, care provider callers and call takers had longer average turn lengths and responded with much fewer one word utterances compared to the older adult. Between low, medium and high risk situations, the average turn lengths did not change across speakers. These results suggest that care provider callers tend to say more than the older adult when it is their turn to speak whereas the older adults seem to respond more via many short utterances.

Greater variability in the average turn lengths for older adult callers was also observed at the medium risk level whereas greater variability was observed at the high risk level for care provider callers. For older adult callers, higher variability in turn length in medium risk situations suggests that different older adult callers may be using different methods of responding to call takers, such as simple one word responses versus longer descriptive responses. However, when the risk becomes high, perhaps there is less description on the part of the older adult and the call taker is able to respond to what is needed fairly quickly (i.e., EMS). The higher variability in medium risk calls may also be reflective of the different kinds of response types being requested. For the care provider caller, higher variability in turn length in high risk situations suggests that different care providers may become more descriptive in their response. It is possible that these response differences may also be because of the type of care provider calling (i.e. novice PERS users or pro PERS user) or the emergency situation (i.e. caller may have a greater need to describe what is happening).

With respect to conversational structure, call takers made fewer statements, asked more questions, and responded to fewer questions, which is in line with the script call takers follow. This script requires call takers to ask mostly close-ended questions until enough information, justification, and verification is obtained to initiate a call response. Although the older adult and care provider callers both conversed using a similar number of statements and questions which was significantly more and less than their call takers, respectively, older adult callers responded to more questions on average than care providers. A possible reason for this is that older adults may be led more in the conversation by the call taker and subsequently responds with simple one word type answers (e.g., yes, no, fine, okay); whereas the care provider may be more direct in responding and provide the necessary information required by the call taker up front. For example, the care provider will often state what (s)he wants and justifies this request, “I need an ambulance to come because Mrs. Smith fell and hit her head and is bleeding”. The questions posed by the call taker may also differ depending on who is calling, the older adult or care provider. The call taker may ask the older adult specific questions about their ailments versus asking a more general overall condition question to a care provider (e.g., Are you hurt? Are you cold? Do you have a temperature?). Depending on who is calling, call takers may also be affected by the authority projected by certain care providers (e.g., a nurse), thus leading to less questioning and a quicker response.

With respect to differences between risk levels, on average, significantly fewer statements were made by older adult callers at high risk levels; however, any differences between risk levels for care provider callers was not significant. In high risk situations with older adults, fewer statements correspond to the finding that older adults also had fewer speaker turns and spoke fewer words. In high risk situations with care providers, their statement results also correspond to the findings that no significance difference was observed in the number of speaker turns at different risk levels. This lack of change in the number of speaker turns at the higher risk level may suggest that care provider callers are already using the average minimum number of speaker turns required before a response can be initiated. Another possibility is that the data results are altered due to the presence of two outliers, #4 and #2 as observed in the care provider high risk category in Fig. 7a. Removing the outliers and re-running the t-test did not change this result (p-value went slightly lower to 0.067). More call samples would be beneficial to strengthen and confirm the result outcomes. Future studies may want to consider examining the speech acts used by different caller types.

In high risk situations, care providers also spoke fewer complete words within a shorter average call response time compared to medium risk situations. This result suggests that even though the desired response of the care provider does not change (EMS (i.e. ambulance) is still desired), (s)he may adjust his/her dialogue to become more succinct when requesting the response. It is also possible that the emergency level of the situation is clear with the particular words used and the call taker responds more quickly. Future studies may look at the lexical semantics used at different risk levels and caller types.

The number of speaker turns required for a call response for the caller group was found to be on average less than the number of speaker turns for the call taker group. This result is not surprising because the call taker generally opens and ends the call. The average number of speaker turns calculated may provide SDS developers of smart PERS a guideline to target with respect to determining how many turns should be permitted before defaulting to a live operator. Between medium and high risk levels, greater variability exists at medium risk levels and more for the older adult callers than care providers. Different turn limits may need to be considered depending on if the speaker is an older adult or care provider caller or high or medium risk.

With respect to timing, in seconds, high risk calls were responded to more quickly than medium risk calls, and no risk calls were identified the fastest. These results did not change between caller types (i.e., older adults and care providers). All calls were less than 3 min in length. The timing results could be used as a baseline for specifying a response time for a smart PERS.

In terms of predicting caller type, the discriminant function analyses revealed that the number of complete words and the average turn length conversational measures would be the best predictors. Using a discriminant function composed of the verbal ability measures: NumWrds, AvgTnLgth, and OneWrdUtts, moderately-high predictive success was obtained with 77.5 % of cases (calls) being correctly classified. Using cross-validation, more older adult calls were correctly classified (85.7 %) than care provider calls (45.5 %). However, this function can be adjusted to favour more care providers than older adults if desired. Repeating the analysis to assess risk level predictability revealed only moderate success with 67.6 % of cases being correctly classified. Using cross-validation, more high risk level calls (63.4 %) were correctly classified than medium level calls (56.3 %). The measure, number of complete words, was the best predictor for risk level.

#### Measures using the Caller’s first spoken turn

Although automatically calculating the number of words and the average turn length of a conversation is possible, waiting until the end of the call would be too late to actually help a dialogue manager tailor its dialogue to a particular caller or risk level. To further examine how the number of complete words spoken might differ between caller types and risk levels, further analyses were performed using only the caller’s first spoken turn.

Analyses using the caller’s first spoken turn revealed that even in this first turn older adult callers spoke significantly more slowly, had significantly shorter turn lengths, and responded using more one word utterances than care provider callers. One explanation for these differences observed in the caller’s first spoken turn is the need for the call taker to get the attention of the older adult caller before to ask if anything is wrong. For example, in some situations, when the PERS is first activated and after the call taker’s first spoken turn, the older adult caller may simply respond with a “hello?” or “yes” (I am here) response. They may not state the reason for the call right away. In contrast, the care provider may respond by not only acknowledging (s)he is present but also stating the reason for the call. For example, “hello, yes, I’m calling about Mrs. Smith, we need an ambulance right away.”

With respect to differences in risk level, neither the older adult nor the care provider callers were found to increase their average first turn words per minute or average first turn length between low, medium or high risk calls. In listening to the call recordings, the older adult callers actually seemed quite calm about their situation. It is unclear whether the older adult callers are simply calm even in high risk situations or whether they want to appear calm so as not to alarm the call taker. The older adult might also be demonstrating that (s)he is in control of the situation. For care provider callers in high risk situations, greater variability was observed for both the first turn length and words per minute measures. This increased variability suggests that some care providers increase their rate of speaking in higher risk situations whereas other care providers may speak more slowly or are distracted when speaking causing them to speak more slowly. In contrast, older adult callers had greater variability in their first turn length and words per minute measures in medium risk situations. This is similar to what was observed for the average turn length measures.

With respect to the number of words used in the first turn length, care providers did not speak more than 40 words with the average being around 15 words. Older adult callers did not speak more than 30 words with the average being 8 words. When designing the ASR component of a SDS for a smart PERS, SDS developers should consider keeping vocabulary size small. Future studies may also want to examine vocabulary differences between callers, risk levels, and situations.

In terms of predicting caller type, the discriminant function analysis revealed that both 1stTnLgth and 1stTnWPM measures were important predictors, however 1^st^TnLgth was the stronger predictive measure. Only moderate predictive success was obtained with 73.2 % of cases being correctly classified. Using cross-validation, more older adult calls were correctly classified (91.8 %) than care provider calls (13.6 %). The low predictive power for care provider callers suggests that the function would need to be adjusted to move the threshold to favour more the care provider caller. It would be interesting to see if recalculating this analysis using the first two or three spoken caller turns would improve the predictive power of these measures.

#### Measuring disfluency

In terms of disfluencies, older adult and care provider callers were found to both have a higher average proportion of maze words compared to the call taker. The higher proportion of mazes for callers may be just a product of having to speak spontaneously in response to the call takers questions and the need to find their words. This is in contrast to the call taker who is generally following scripted dialogue during the conversation. The proportion of maze words per total words was found to be lower for the care provider than the older adult, although this was not significant. It is possible that with more data samples a significant difference would be observed. In Fig. 4d, case 47 was found to have a very high number of maze words, 48 %. A closer examination of this case revealed that the caller had a significant speech impediment which resulted in a great deal of stuttering. In situations with many maze words, it may be difficult for an SDS to decipher what an individual is saying. If these situations could be identified early on in the conversation, the smart PERS could automatically default to a live call taker. Future work might determine how often maze words occur within the initial speaker turns of the response call conversation and whether the proportion of maze words would be representative of the rest of the conversation. However, whether maze words could actually be identified automatically by a computer may difficult. A concern would be how the computer could differentiate between an out of vocabulary word versus a maze word or unintelligible word. What might be possible is if word repetition is measured, for example, “I I I I want…”. Pausing may also be an indication of something wrong, for example “I don’t {pause} I don’t {pause} feel {pause} so good.” In situations where the SDS cannot understand anything, it may just be best to default to a live call taker. If the smart PERS was part of a smart home system with cameras, the cameras’ input could also shed light on if a person was in trouble or not (e.g. present but not moving on floor).

Note that ‘speaker intelligibility’ was excluded from this analysis. Upon close examination of the recorded transcripts, it was difficult to determine true unintelligibility in many situations due to recording issues (e.g., two speakers speaking concurrently; call taker’s voice being recorded directly at the microphone versus the caller’s being transmitted over speaker phone). Future research may want to consider examining speech intelligibility measures if better call recordings can be obtained.

In this study prosody was also not examined. Several papers have found that measures such as jitter and shimmer can be used to identify older adult callers [51, 102]. It is unclear whether the recording quality of calls would have been sufficient to do this type of analysis. Although the recording of the call taker was fairly clear, the recording of the callers was not always clear. Future studies might look into examining prosody in PER call conversations. Perhaps calculating rate of speech, number of words, and jitter and shimmer for incoming speech would be enough to help a SDS identify the caller type with higher probability.

#### General discussion

If a SDS is able to classify an incoming call, this may help the system to predict with some probability the final call response. Hypothetically, if the prediction is correct and call dialogue is adapted to the end-user, the call may be more quickly resolved without the end-user experiencing any SDS frustrations. In order for a computer to automatically classify a call, it is necessary to examine how different callers respond in PER events and to determine a good way to differentiate calls. If caller type or risk level can be predicted with relatively high probability, for example, by counting the number of words spoken or calculating the rate of speech during the initial spoken caller turn(s), this may be a fairly good way to help the decision manager determine next steps. A possible dialogue option for a SDS in a smart PERS may be a routine to verify whether the system has gained the caller’s attention or to recover from silent responses or unintelligible responses. Then, depending on who the caller is, it is possible that none of these options may be necessary or all of the options might be needed. For example, if the caller is predicted to be a care provider, these optional routines might be bypassed and the SDS might simply offer an ambulance response and ask for confirmation. If the caller is an older adult, it may be necessary to grab his/her attention, assess the possible risk level, and offer an ambulance for high risk situations or a known responder or call taker at the medium risk level. The maximum time limit for a response to be identified before defaulting to a live call taker might also be modified depending on the call type (i.e., medium versus high risk).

For the hopefully rare event where a true emergency is ‘missed’ (false negative), the smart PERS system should be designed robustly enough to allow the user to either reactivate the system or if the user is immobile, the system should reactivate itself after no input is received. This missed emergency ‘error’ may cost valuable time, however, if the individual who needs help eventually gets help by having this system, then it is probably better than the situation where the individual does not have a PERS or has one but is unable to push the button and cannot get help.

#### Study limitations

This study was limited by its small and unbalanced sample size. Increasing the sample size may improve the robustness of the results. Also, using a different definition for a ‘fall call’, may increase the number of ‘call reason – fall call’ events and allow this category to be included in the MANOVA analysis. The fact that all response call recordings had come from a single PERS provider also limits the number of PESs represented in this study and the generalizability of the findings. Other PERS providers may follow different call protocols and may experience other types of events which were not observed with the PERS provider where the calls examined were obtained. Another study limitation is transcription variability resulting from human error (e.g., difficulty hearing call recordings clearly). In addition, the fact that statistical analyses are based on mean measurements is also a limitation. Wide variances in measures were observed for both caller and speaker types and simply looking at means does not provide a complete picture of what may be happening within each call. Finally, call meta-data surrounding the speaker details was not provided (e.g., which call taker is responding, gender of callers, caller medical history). As such, this study is limited in its ability to define further sub-groups of callers or call reasons.

## Conclusions

This study demonstrates how mixed-methods could be applied to analyze secondary data, real recorded PER calls, for the purpose of identifying contextual information about PERS end-users and PER events for inclusion into the design of a SDS in a smart PERS. Specifically, this data could be applied to help the SDS identify, to moderate probability, the caller type within the first spoken turn of the caller, guide prediction of the target response (response type) based on identified caller type, and provide benchmarks for maximum conversation time.

In summary, the qualitative analysis identified two main call response types including EMS and non-EMS responses. Calls initiated by care providers resulted in an EMS response 100 % of the time. Calls initiated by older adults resulted in EMS response nearly 96 % of the time in high risk situations and 71 % of the time in medium risk situations. For older adult callers, the use of the word “ambulance” versus “paramedic” may be significant and consideration should be given as to when and how these terms are used when proposing a call response. For example, the term “ambulance” might be used in high risk situations or with care provider callers and “paramedic” might be used in medium risk situations or with older adult callers.

The quantitative analyses examined the relationships between several conversational measures and caller type and risk level, and in some cases speaker type (callers versus call takers). Initial analysis revealed that the caller’s number of complete words and their average turn length could be useful in predicting caller type and risk level. Subsequent analyses focused only on the first turn length and first turn words per minute and demonstrated that caller type only could be predicted with moderate success. Older adults were also shown to respond more with one word utterances during the first spoken turn than care providers. It would be interesting to see if using several initial speaker turns rather than just the first turn would further strengthen caller type identification or improve risk level estimation. Given the lower probability for estimating risk level, the ability for the SDS to estimate risk level may need to rely heavily on understanding the lexical semantics of the spoken speech.

With respect to conversational patterns, care provider and older adult callers were shown to employ different strategies for responding to call takers. Care providers seemed to say more in fewer speaker turns whereas older adult callers tended to say less in a speaker turn and required more turns. Variability in turn length was also higher in medium risk calls for the older adult callers than high risk calls, but reversed for care providers who had higher variability in high risk calls and lower in medium risk calls. In listening to the calls, in high risk situations, older adults seemed fairly calm in requesting assistance whereas some care provider callers seemed more panicked or stressed in high risk situations. Future studies may want to verify this observation by measuring stress in the voice or subjectively with different raters.

Looking at call timing, the study identified values for average call taker response times in both speaker turns and in seconds. These values may be useful as a baseline for managing a smart PERS automated dialogue response and setting the maximum time allowed for a conversation at various risk levels before defaulting to a live operator.

In conclusion, if the call pattern information and conversational measures are combined with the ASR and natural language processing outputs (lexical semantics), the resulting SDS may be able to identify PERS caller type and/or risk level with greater confidence and subsequently predict a target response. With a possible target response identified, the SDS can tailor the output dialogue to the caller type. We hypothesize these study results will help improve the artificial intelligence and decision making ability of a SDS in a smart PERS and that doing so will help the system respond well to different caller types and risk levels, subsequently identifying the desired response type more quickly (increase efficiency). A well-functioning SDS will provide a good user experience (decrease user frustration) and theoretically, lead to higher PERS usage rates and lower technology abandonment.

## Abbreviations

1stTnLgth: Caller’s first spoken turn length in words
1stTnWPM: Caller’s first spoken turn words per minute
ASR: Automatic speech recognition
AvgTnLgth: Average turn length in words
EMS: Emergency medical services
HELPER: Health Evaluation Logging and Personal Emergency Response
NumQues: Number of questions
NumResQues: Number of responses to questions
NumSpkrTns: Speaker turns
NumStmt: Number of statements
OneWrdUtts: Number of one word utterances
PER: Personal emergency response
PERS: personal emergency response system
PES: Personal emergency situations
PrctMazes: Proportion of total words with mazes
SALT: Systematic Analysis of Language Transcripts
StdDev: Standard deviation

## References

[1] Lee T, Mihailidis A (2005). An intelligent emergency response system: preliminary development and testing of automated fall detection. J Telemed Telecare.

[2] Belshaw M, Taati B, Snoek J, Mihailidis A. Towards a single sensor passive solution for automated fall detection. In Proceedings of the 2011 International Conference of the IEEE Engineering in Medicine and Biology Society (EMBS). 2011. p. 1773–6. doi:10.1109/IEMBS.2011.6090506. 10.1109/IEMBS.2011.6090506 PMC3465367 22254671

[3] Hamill M, Young V, Boger J, Mihailidis A (2009). Development of an automated speech recognition interface for personal emergency response systems. Master’s Thesis. J Neuroeng Rehabil.

[4] McLean MH. Design of a Speech Recognition Interface for a Personal Emergency Response and Health Monitoring System. Master’s Thesis. University of Toronto; 2005.

[5] Dibner AS (1993). Personal response services present and future. Home Health Care Serv Q.

[6] Hessels V, Le Prell GS, Mann WC (2011). Advances in personal emergency response and detection systems. Assist Technol.

[7] Mann W, Belchior P, Tomita MR, Kemp BJ (2005). Use of personal emergency response systems by older individuals with disabilities. Assist Technol.

[8] Montgomery C (1993). Personal response systems in the United States. Home Health Care Serv Q.

[9] Roush RE, Teasdale TA, Murphy JN, Kirk MS (1995). Impact of a personal emergency response system on hospital utilization by community-residing elders. South Med J.

[10] Porter EJ, Ganong LH (2002). Considering the use of a personal emergency response system: an experience of frail, older women. Care Manag J.

[11] Heinbüchner B, Hautzinger M, Becker C, Pfeiffer K (2010). Satisfaction and use of personal emergency response systems. Zeitschrift fuer Gerontologie und Geriatrie.

[12] Taylor A, Agamanolis S. Service users’ views of a mainstream telecare product: the personal trigger. In Proceedings of the 28th International ACM Conference Extended Abstracts on Human Factors in Computing Systems (CHI). Atlanta; 2010. p. 3259–64. doi:10.1145/1753846.1753968.

[13] Porter EJ (2005). Wearing and using personal emergency response system buttons. J Gerontol Nurs.

[14] Fleming J, Brayne C (2008). Inability to get up after falling, subsequent time on floor, and summoning help: prospective cohort study in people over 90. BMJ.

[15] Blythe MA, Monk AF, Doughty K (2005). Socially dependable design: the challenge of ageing populations for HCI. Interacting Comput.

[16] Davies KN, Mulley GP (1993). The views of elderly people on emergency alarm use. Clin Rehabil.

[17] Fallis WM, Silverthorne D, Franklin J, McClement S (2007). Client and responder perceptions of a personal emergency response system: Lifeline. Home Health Care Serv Q.

[18] Demiris G, Hensel B, Skubic M, Rantz M (2008). Senior residents’ perceived need of and preferences for “smart home” sensor technologies. Int J Technol Assess Health Care.

[19] Chan M, Campo E, Estève D, Fourniols J-Y (2009). Smart homes—current features and future perspectives. Maturitas.

[20] Doughty K, Cameron K, Garner P (1996). Three generations of telecare of the elderly. J Telemed Telecare.

[21] Anderson S, Liberman N, Bernstein E, Foster S, Cate E, Levin B, Hudson R. Recognition of elderly speech and voice-driven document retrieval. In 1999 Proceedings IEEE International Conference on Acoustics, Speech, and Signal Processing. 1999;1:145–8.

[22] Vacher M, Lecouteux B, Portet F. On Distant Speech Recognition for Home Automation. In: Holzinger A, Röcker C, Ziefle M, editors. Lecture Notes in Computer Science, Smart Health: Open Problems and Future Challenges. Switzerland: Springer International Publishing. 2015;8700:161–88.

[23] Mӧller S, Gӧdde F, Wolters M. A corpus analysis of spoken smart-home interactions with older users. In Proceedings of the 6th International Conference on Language Resources and Evaluation (LREC). Marrakech; 2008. p. 735–40.

[24] López-Cózar R, Callejas Z, Griol D, Quesada JF (2015). Review of spoken dialogue systems. Loquens.

[25] Johnson JL, Davenport R, Mann WC (2007). Consumer feedback on smart home applications. Topics Geriatr Rehabil.

[26] Portet F, Vacher M, Golanski C, Roux C, Meillon B (2013). Design and evaluation of a smart home voice interface for the elderly: acceptability and objection aspects. Pers Ubiquitous Comput.

[27] Chen XY, Zang J, Yang M, Wang WP, Hu Y (2014). Design and development of self-help emergency device based on the android intelligence platform. Appl Mech Mater.

[28] Lamel L, Minker W, Paroubek P (2000). Towards best practice in the development and evaluation of speech recognition components of a spoken language dialog system. Nat Lang Eng.

[29] Georgila K, Wolters M, Moore JD, Logie RH (2010). The MATCH corpus: a corpus of older and younger users’ interactions with spoken dialogue systems. Lang Res Eval.

[30] Mӧller S. Quality of Human-Machine Interaction over the Phone. In: Quality of Telephone-Based Spoken Dialogue Systems. India: Springer US; 2005. p. 9–91. doi:10.1007/b100796.

[31] Kim Y-U, Kang S-K, So I-M, Han D-K, Lee S-S, Lee Y-J, Jung S-T. Emergency recognition system based on multimodal information. In Proceedings of the 30th Annual International Conference of the IEEE Engineering in Medicine and Biology Society (EMBS). Vancouver; 2008. p. 4342–45. 10.1109/IEMBS.2008.4650171 19163674

[32] Vacher M, Istrate D, Portet F, Joubert T, Chevalier T, Smidtas S, Meillon B, Lecouteux B, Sehili M, Chahuara P, et al. The sweet-home project: Audio technology in smart homes to improve well-being and reliance. In Proceedings of the 2011 International Conference of the IEEE Engineering in Medicine and Biology Society (EMBS). 2011. p. 5291–94. 10.1109/IEMBS.2011.6091309 22255532

[33] Bouakaz S, Vacher M, Chaumon M-EB, Aman F, Bekkadja S, Portet F, Guillou E, Rossato S, Desserée E, Traineau P (2014). CIRDO: smart companion for helping elderly to live at home for longer. IRBM.

[34] Eskenazi M. Trends in speaking styles research. In Proceedings of the Third European Conference on Speech Communication and Technology (EUROSPEECH). Berlin: ISCA; 1993.

[35] Sikorski W (2012). Paralinguistic communication in the therapeutic relationship. Arch Psychiatry Psychother.

[36] Newell AF, Gregor P (2000). “User sensitive inclusive design”—in search of a new paradigm. Proceedings on the 2000 Conference on Universal Usability (CUU).

[37] Gregor P, Newell AF, Zajicek M. Designing for dynamic diversity: interfaces for older people. In Proceedings of the Fifth International ACM Conference on Assistive Technologies (Assets). Edinburgh; 2002. p. 151–6.

[38] Vacher M, Caffiau S, Portet F, Meillon B, Roux C, Elias E, Lecouteux B, Chahuara P. Evaluation of a context-aware voice interface for ambient assisted living: qualitative user study vs. quantitative system evaluation. ACM Trans Accessible Comput (TACCESS). 2015;7(2, Article 5):1–36.

[39] Vipperla R, Wolters M, Georgila K, Renals S. Speech input from older users in smart environments: Challenges and perspectives. In Proceedings of the Fith International Conference of Universal Access in Human-Computer Interaction (UAHCI) with HCI International, Intelligent and Ubiquitous Interaction Environments. San Diego: Springer. 2009. p. 117–26. (Part II).

[40] Lippmann RP (1997). Speech recognition by machines and humans. Speech Commun.

[41] Baba A, Yoshizawa S, Yamada M, Lee A, Shikano K (2004). Acoustic models of the elderly for large-vocabulary continuous speech recognition. Electron Commun Japan.

[42] Wilpon JG, Jacobsen CN. A study of speech recognition for children and the elderly. In Proceedings of the International Conference of the IEEE Acoustics, Speech, and Signal Processing (ICASSP). 1996;1:349–52.

[43] Yorkston KM, Bourgeois MS, Baylor CR (2010). Communication and aging. Phys Med Rehabil Clin N Am.

[44] Devillers L, Vidrascu L. Real-life emotion recognition in speech. In: Müller C, editor. Lecture Notes in Computer Science, Speaker Classification II - Selected Projects. Berlin Heidelberg: Springer. 2007;4441:34–42.

[45] Fogle CC, Oser CS, Troutman TP, McNamara M, Williamson AP, Keller M, McNamara S, Helgerson SD, Gohdes D, Harwell TS (2008). Public education strategies to increase awareness of stroke warning signs and the need to call 911. J Public Health Manag Pract.

[46] Handschu R, Poppe R, Rauss J, Neundörfer B, Erbguth F (2003). Emergency calls in acute stroke. Stroke.

[47] LaPointe LL. Chapter 9: Neurogenic disorders of communication. In: Minifie, FD, editor. Introduction to communication sciences and disorders. New York: Delmar Cengage Learning; 1994. p. 351–97.

[48] Hansen JH, Patil S. Speech under stress: Analysis, modeling and recognition. In: Müller, C, editor. Speaker Classification I: Fundamentals, Features and Methods, Lecture Notes in Computer Science. Berlin Heidelberg: Springer. 2007;4343:108–37.

[49] Orange J (2009). Language and communication disorders in older adults: selected considerations for clinical audiology.

[50] Edwards S, Tucker K (2002). Presentation: Communication problems in the elderly.

[51] Müller C, Wittig F, Baus J. Exploiting speech for recognizing elderly users to respond to their special needs. In Proceedings of the Eight European Conference on Speech Communication and Technology (EUROSPEECH and INTERSPEECH). Geneva: ISCA; 2003, 3:1305–8.

[52] Lefter I, Rothkrantz LJ, Van Leeuwen DA, Wiggers P (2011). Automatic stress detection in emergency (telephone) calls. Int J Intell Def Support Syst.

[53] Demenko Grazyna, Jastrzebska M. Analysis of voice stress in call centers conversations. In Proceedings of Speech Prosody 6th International Conference. Shanghai; 2012.

[54] Furui S. Toward robust speech recognition and understanding. In: Matousek V and Mautner P, editors. Lecture Notes in Artificial Intelligence, Proceedings of Text, Speech and Dialogue (TSD) 6th International Conference. Ceské Budejovice, Czech Republic. Springer-Verlag Berlin Heidelberg; 2003, 2807:2–11.

[55] Takahashi S, Morimoto T, Maeda S, Tsuruta N. Robust speech understanding based on expected discourse plan. In Proceedings of the Eight European Conference on Speech Communication and Technology (EUROSPEECH) and INTERSPEECH. Geneva: ISCA; 2003.

[56] Furui S. Spontaneous speech recognition and summarization. In: Langemets M, Penjam P, editors. Proceedings of the Second Baltic Conference on Human Language Technologies. Tallinn: ISCA; 2005, 39:50–61.

[57] Cromdal J, Osvaldsson K, Persson-Thunqvist D (2008). Context that matters: producing “thick-enough descriptions” in initial emergency reports. J Pragmat.

[58] Garcia AC, Parmer PA (1999). Misplaced mistrust: the collaborative construction of doubt in 911 emergency calls. Symbolic Interact.

[59] Garner M, Johnson E (2007). Operational communication: a paradigm for applied research into police call-handling. Int J Speech Lang Law.

[60] Imbens-Bailey A (2000). The discourse of distress: a narrative analysis of emergency calls to 911. Lang Commun.

[61] Waseem H, Durrani M, Naseer R (2010). Prank calls: a major burden for an emergency medical service. Emerg Med Australas.

[62] Whalen M, Zimmerman D. Sequential and institutional contexts in calls for help. Social Psychology Quarterly. 1987;50(2):172–85.

[63] Plano Clark VL, Creswell JW. Chapter 3: Choosing a mixed methods design. In Designing and conducting mixed methods research. 2nd ed. Thousand Oaks, California: Sage Publications; 2011:53–106.

[64] Krippendorff K. Content analysis: An introduction to its methodology. Second ed. Thousand Oaks, California: Sage Publications; 2012.

[65] Cavanagh S (1997). Content analysis: concepts, methods and applications. Nurse Res.

[66] Elo S, Kyngäs H (2008). The qualitative content analysis process. J Adv Nurs.

[67] Polit DF, Beck CT. Nursing research: Principles and methods. 7th edition. Philadelphia: Lippincott Williams & Wilkins; 2004.

[68] Graneheim UH, Lundman B (2004). Qualitative content analysis in nursing research: concepts, procedures and measures to achieve trustworthiness. Nurse Educ Today.

[69] Sidnell J, Stivers T. The Handbook of Conversation Analysis. Chichester: Wiley-Blackwell; 2012.

[70] Hsieh H-F, Shannon SE (2005). Three approaches to qualitative content analysis. Qual Health Res.

[71] Morgan DL (1993). Qualitative content analysis: a guide to paths not taken. Qual Health Res.

[72] Mondada L. The conversation analytic approach to data collection. In: Sidnell J, Stivers T, editors. The Handbook of Conversation Analysis. Chichester: Wiley-Blackwell; 2012. p. 32–56.

[73] Teas Gill V, Roberts F. Conversation analysis in medicine. In: Sidnell J, Stivers T, editors. The Handbook of Conversation Analysis. Chichester: Wiley-Blackwell; 2012. p. 575–92.

[74] Toronto Paramedic Services [http://www1.toronto.ca/wps/portal/contentonly?vgnextoid=74338d5c19c52410VgnVCM10000071d60f89RCRD]. Accessed 17 May 2016.

[75] Breen T (1993). Community alarm systems. Home Health Care Serv Q.

[76] De San MK, Lewin G (2008). Brief report: personal emergency alarms: what impact do they have on older people’s lives?. Australas J Ageing.

[77] Miller J, Iglesias A. Systematic Analysis of Language Transcripts (SALT), English & Spanish (Version 9) [Computer Software]. Middleton: Language Analysis Lab, University of Wisconsin-Madison; 2006.

[78] Audacity Team. Audacity®. Version 2.0.2. A free digital audio editor. [http://audacity.sourceforge.net]. 2012. Accessed 1 Dec 2012.

[79] Miller J, Chapman RS. Systematic Analysis of Language Transcripts (SALT), English (Version 8) [Computer Software]. Middleton: Language Analysis Lab, University of Wisconsin-Madison. 2005.

[80] IBM Corp. IBM SPSS Statistics for Windows, Version 22.0. Armonk: IBM Corp. Released 2013.

[81] Young V. Content Analyses of Personal Emergency Response Calls: Towards a More Robust Spoken Dialogue-Based Personal Emergency Response System. Doctoral Thesis. University of Toronto; 2016.

[82] Gilboy N, Tanabe P, Travers D, Rosenau A, Eitel D, et al. Emergency severity index, version 4: implementation handbook. Rockville: Agency for Healthcare Research and Quality; 2005. p. 1–72.

[83] Manitoba Education Training and Youth. Severity Rating: Functional Communication Measures. In: Manitoba Speech-Language Pathology Outcome Measures: An implementation manual for speech-language pathologists and administrators. Manitoba, Saskatchewan; 2000.

[84] Sacks H, Schegloff EA, Jefferson G. A simplest systematics for the organization of turn-taking for conversation. Language. 1974;50(4, Part I):696–735.

[85] Crookes G (1990). The utterance, and other basic units for second language discourse analysis. Appl linguist.

[86] Culatta R, Leeper LH (1990). The differential diagnosis of disfluency. Natl Stud Speech Lang Association J.

[87] Hall N, Wagovich SA, Bernstein Ratner N: Chapter 9 - Language considerations in childhood stuttering, Stuttering and related fluency disorders. In: Conture E, Curlee R, editors. Third ed. Stuttering and related fluency disorders. New York: Thieme; 2007. p. 153–67.

[88] Leadholm BJ, Miller J. Language Sample Analysis: The Wisconsin Guide. Madison: Wisconsin Department of Public Instruction; 1995.

[89] Bortfeld H, Leon SD, Bloom JE, Schober MF, Brennan SE (2001). Disfluency rates in conversation: effects of age, relationship, topic, role, and gender. Lang Speech.

[90] Fox Tree JE (1995). The effects of false starts and repetitions on the processing of subsequent words in spontaneous speech. J Mem Lang.

[91] Shriberg EE. Phonetic consequences of speech disfluency. In Proceedings of the 14th International Congress of Phonetic Sciences (ICPhS). San Francisco; 1999. p. 619–22.

[92] Zraick RI, Gregg BA, Whitehouse EL (2006). Speech and voice characteristics of geriatric speakers: a review of the literature and a call for research and training. J Med Speech Lang Pathol.

[93] Yuan J, Liberman M, Cieri C. Towards an integrated understanding of speaking rate in conversation. In Proceedings of the Ninth International Conference on Spoken Language Processing (INTERSPEECH-ICSLP). Pittsburgh; 2006. p. 1–4.

[94] Private_PERS_Call_Centre. Operations Protocol for PERS Call Centre. Canada, 2008.

[95] Silverman RA, Galea S, Blaney S, Freese J, Prezant DJ, Park R, Pahk R, Caron D, Yoon S, Epstein J (2007). The “vertical response time”: barriers to ambulance response in an urban area. Acad Emerg Med.

[96] Pons PT, Haukoos JS, Bludworth W, Cribley T, Pons KA, Markovchick VJ (2005). Paramedic response time: does it affect patient survival?. Acad Emerg Med.

[97] Mullie A, Van Hoeyweghen R, Quets A (1989). Influence of time intervals on outcome of CPR. Resuscitation.

[98] Eisenberg MS, Bergner L, Hallstrom A (1979). Cardiac resuscitation in the community. JAMA.

[99] Blackwell TH, Kaufman JS (2002). Response time effectiveness: comparison of response time and survival in an urban emergency medical services system. Acad Emerg Med.

[100] Hyer K, Rudick L (1994). The effectiveness of personal emergency response systems in meeting the safety monitoring needs of home care clients. J Nurse Adm.

[101] Field A. Discovering statistics using SPSS. 2nd ed. London: Sage publications; 2005.

[102] Dehqan A, Scherer RC, Dashti G, Ansari-Moghaddam A, Fanaie S (2013). The effects of aging on acoustic parameters of voice. Folia Phoniatr Logop.

